# Representational shifts: increasing motivation for bottled water through simulation-enhancing advertisements

**DOI:** 10.1186/s12889-023-17109-1

**Published:** 2023-11-09

**Authors:** Maria Almudena Claassen, Esther Katharina Papies

**Affiliations:** 1 Center for Adaptive Rationality, Max-Planck-Institute for Human Development, Lentzeallee 94, 14195 Berlin, Germany; 2 School of Health & Wellbeing, University of Glasgow, 90 Byres Road, Glasgow, G12 8TB UK

**Keywords:** Water consumption, Reward, Advertisements, Health intervention

## Abstract

**Background:**

Despite its numerous health benefits, consumers’ daily water consumption is below recommend levels while soft drink consumption remains high. Previous research has shown that the degree to which drinks are cognitively represented in terms of consumption and enjoyment (i.e., through simulations of consumption and reward) predicts desire and intake. Here, we examined whether simulation-enhancing advertisements that frame water in terms of consumption and reward change cognitive representations and increase motivation for a fictitious bottled water.

**Methods:**

In three pre-registered online experiments (*N*_exp1_ = 984; *N*_exp2_ = 786; *N*_exp3_ = 907), UK participants viewed three advertisements that either highlighted the rewarding consumption experience of water (e.g., “Refresh all your senses with this smooth, cool water”; simulation-enhancing ads), the health consequences of drinking water (e.g., “This water takes care of your health”; health-focused ads), or control ads. We assessed cognitive representations of the bottled water with a semantic feature production task, and we coded the words used as consumption and reward features or positive long-term health consequences features. We assessed motivation through ratings of the attractiveness of the water (Exp. 1 only), desire to drink it, and willingness to pay for it (WTP).

**Results:**

In line with our hypotheses, participants represented the bottled water more in terms of consumption and reward after viewing simulation-enhancing advertisements, and more in terms of positive long-term health consequences after viewing health-focused advertisements. There was no direct effect of advertisement condition on motivation ratings. However, significant indirect effects showed that simulation-enhancing advertisements increased desire and WTP through the proportion of consumption and reward features, whereas health-focused advertisements increased motivation through an increase in the proportion of positive long-term health consequences features. The effects through consumption and reward were stronger.

**Conclusions:**

These findings are consistent with research suggesting that the experience of immediate reward from drinking water underlies intake. Public health interventions should emphasize the enjoyment of drinking water, rather than the long-term health benefits.

**Supplementary Information:**

The online version contains supplementary material available at 10.1186/s12889-023-17109-1.

## Background

The consumption of sugar-sweetened beverages (SSBs) is associated with adverse health outcomes such as metabolic disease and poor dental health [1, 2]. In contrast, maintaining healthy hydration through water consumption is associated with improved physical and cognitive function [3, 4]. On average, people do not reach the recommended amount of daily fluid intake [5], and less than half of this amount comes from plain water [6]. Public health interventions aimed at reducing SSB consumption or increasing water consumption include levying sugar taxes, educational interventions, or environmental interventions such as restructuring the choice environment [7, 8].

Taxes on SSBs have been somewhat successful in reducing sugar intake [9] and have been associated with reduced obesity rates [10]. However, reducing SSB intake does not necessarily increase the availability or consumption of water or other non-sweetened beverages. Only small increases in water consumption have been observed through environmental restructuring in combination with an educational intervention, and these approaches are expensive and time-consuming [11].

One possible reason for the relative ineffectiveness of current interventions is the limited understanding of what drives motivation to drink water. Recent research suggests that the way people cognitively represent water and other drinks may underlie their drinking motivation. Specifically, a series of experiments assessing cognitive representations, desire, and intake showed that motivation for bottled water and other drinks was predicted by representing them through re-experiences of drinking and enjoying them [12, 13]. Representing drinks in terms of consumption and reward predicted motivation and intake more strongly than representing them in terms of their long-term health consequences [12]. In other words, mentally simulating the rewarding act of consuming water can evoke thoughts about the taste, mouthfeel, and energizing effects of water, which in turn predicts motivation to drink it, more so than thinking about its health benefits. These findings are consistent with the grounded cognition theory of desire and motivated behavior, which suggests that consumption experiences of appetitive stimuli are stored in a multimodal memory representation, as a situated conceptualization [14]. When people later encounter the stimuli again, the memory is activated and the consumption experience that is stored in these multimodal representations, is reexperienced or simulated, which in turn may guide motivation [15]. Indeed, when people spontaneously thought about immediate sensory (e.g., cold) or rewarding (e.g., refreshing) properties of water, they were more likely to desire it and drank more of it [12, 16]. Similarly, alcoholic drinks and SSBs were heavily described using words related to sensory aspects and the consumption context [17]. Hence, the way drinks are cognitively represented may play an important role in drinking behavior.

Previous research in the domain of food has shown that framing healthy foods in terms of their sensory and rewarding properties is effective at increasing desire for and consumption of these foods [18, 19]. This approach increases the expected and even actual reward obtained when consuming the foods [20, 21]. Applying this to the domain of drinks suggests that changing cognitive representations of drinks may be an effective strategy for changing drinking behavior. In particular, making consumption and reward features of water more salient may increase the motivation to drink water, possibly more so than focusing on its health benefits, as is often done in water advertising.

In a series of experimental studies building on this line of reasoning, we recently tested whether using sensory and reward-related language on bottled water labels could increase the appeal of water [13]. While we acknowledge the environmental implications of plastic water bottles, focusing on bottled water may still be beneficial in consumption situations in which bottled water competes with more rewarding bottled drinks such as SSBs, which are often advertised in terms of reward, as seen in vending machines and fast food restaurants [22, 23]. The results of these experiments showed that labels enhanced with sensory and reward language (e.g., “Indulge in refreshment: A cool splash of St. Moulin water will instantly restore your body and mind”) increased ratings of attractiveness, anticipated reward, and desire for water compared to labels with only a brand name. However, the effects were similar in strength to those of conventional labels referencing the origin and purity of the water (e.g., “St. Moulin brings you water from organic plains of Tuscany. Original in nature”; 13). This suggests that emphasizing the rewarding aspects of water may be an effective way to increase the appeal of bottled water, but that this strategy can be improved. Possibly, using both images and words that emphasize consumption and reward experiences of drinking water can more effectively shift representations of water to be more rewarding in the moment, and hence increase motivation to drink it.

In addition, our previous findings [13] also revealed an indirect effect, namely that desire for the water increased through increases in the expected reward of the bottled water, after viewing bottled water labels enhanced with sensory and reward language compared to brand-only labels. This is consistent with research highlighting the impact of extrinsic cues, such as a product’s packaging, on shaping expectations of liking, thereby influencing both consumption motivation and the subsequent consumption experience, possibly through the activation of reward areas in the brain [21]. For example, increasing the weight of a product affects desire and Willingness To Pay (WTP) due to increased perceptions of flavor intensity [24]. These findings suggest that motivation for food or drinks may be driven by changes in expected reward in response to product cues.

## Overview

We tested whether increasing the salience of different properties of bottled water in image-based advertisements can shift people’s representations of a bottled water, and increase ratings of reward, health, and motivation for this water. In a series of three experiments, participants were invited to view advertisements of a fictitious bottled water that emphasized either sensory and reward features (simulation-enhancing ads) or features related to the health benefits of water (health-focused ads), or an unrelated advertisement (control ads). In the first two experiments, participants were instructed to immerse themselves for 30s in the displayed advertisements. Previous research has shown that mentally immersing oneself in food images increases the experience of eating simulations [25], and that imagining the rewarding or sensory properties of food increases anticipated reward [26]. Then, in Exp. 3, we examined whether viewing the advertisements in a more naturalistic manner for a shorter period of time and without immersive instructions would lead to the same effects.

After participants viewed the advertisements, we assessed cognitive representations of the bottled water with a Feature Listing task, a semantic feature production task commonly employed in cognitive science to tap into mental representations [﻿^27^]. In this task, participants are asked to list “typical features” or characteristics of a concept, or simply to describe it [28, 29], after which the features produced are coded into different categories of interest to the researchers. This has previously been used to study the cognitive representations of various food and drink products, showing for example that appealing foods and drinks are more likely to be described in terms of consumption experiences than less appealing foods and drinks [17, 28, 29]. Here, this task allowed us to measure the extent to which the advertisements led participants to represent the bottled water in terms of consuming and enjoying it, or in terms of longer-term health outcomes.

**Fig. 1:**
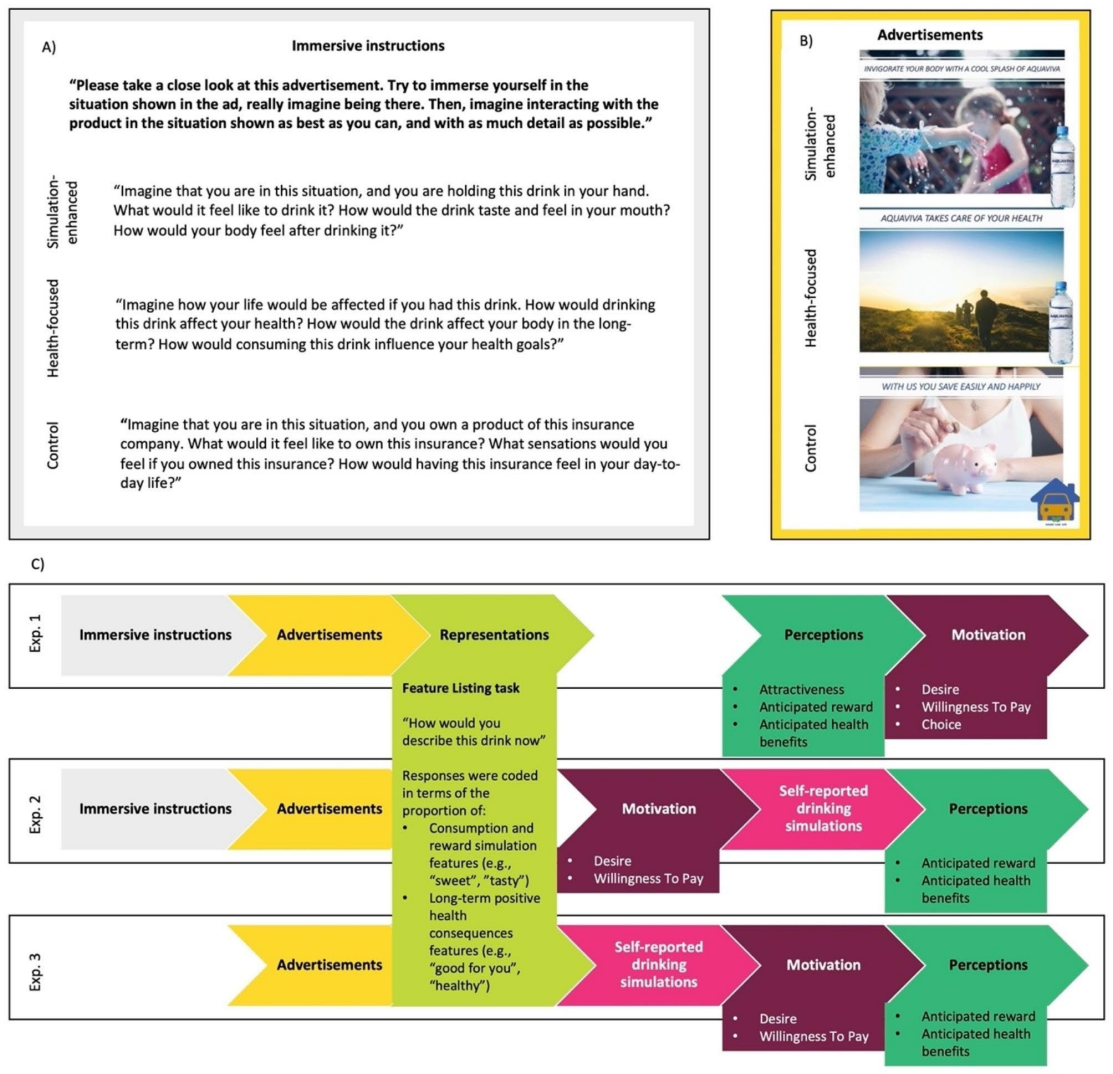
Description of Exp. 1–3. (**A**) Immersive instructions shown alongside the advertisements in Exp. 1 and 2. (**B**) Examples of advertisements used. See Figs. S1, S5, and S8 in the SOM for the complete sets of slogans used. The actual advertisements used can be requested from the authors. (**C**) Procedure of the three experiments. In Exp. 1, we assessed the impact of advertisements on perceptions and motivation. Since we found no significant effect of advertisements on motivation, we assessed motivation before perceptions in Exp. 2. In addition, we assessed drinking simulations in Exp. 2. Then, in Exp. 3, we assessed drinking simulations before motivation and perceptions for a more direct test of the serial mediation

In addition, we used rating scales to assess anticipated reward and anticipated health benefits, as well as general perceptions of attractiveness of the bottled water (only in Exp. 1). In addition, motivation to drink the bottled water was assessed through ratings of desire and WTP, which assesses the maximum amount a person is willing to spend for a product [24]. In addition, motivation was assessed in Exp. 1 through a hypothetical choice task as a proxy of actual behavioral choice. In summary, this series of experiments allowed us to examine the association between different patterns of cognitive representations of water, as assessed with the feature production task, with perceptions of attractiveness, reward and health, and motivation to drink, assessed with rating scales. See Fig. 1 for an overview of the experimental methodology.

## Experiment 1

In Exp. 1, we tested the hypotheses that simulation-enhancing advertisements would increase representations of consumption and reward (Hyp. 1), and ratings of anticipated reward (Hyp. 2), attractiveness (Hyp. 3) and desire (Hyp. 4) for the bottled water compared to health-focused and control advertisements. In addition, we predicted that simulation-enhancing advertisements would increase the attractiveness of water compared to health-focused or control advertisements through increased anticipated reward (mediation; Hyp. 3b), and we predicted the same pattern for desire (mediation; Hyp. 4b). Finally, we predicted that simulation-enhancing advertisements would increase the choice of the advertised bottled water over a Coca-Cola compared to health-focused and control advertisements (Hyp 5.). In addition, we explored whether simulation-enhancing advertisements would increase WTP, whether health-focused advertisements would increase anticipated health benefits and cognitive representations in terms of positive long-term health consequences, and whether cognitive representations of consumption and reward and positive long-term health consequences would mediate the effect of advertisement condition on motivation for water as assessed by desire and WTP.

### Method

#### Participants

Power analyses suggested a minimum sample size of *N* = 975 (see the SOM for more details). Participants were invited to participate through the online platform Prolific [30] if they (1) lived in the UK, (2) were between 18 and 70 years old, and (3) had not participated in any of our previous studies on beverage consumption. We collected data from *N* = 989 participants. Based on our pre-registered exclusion criteria, we excluded participants who took more than four times the median time to complete the study (*n* = 5). The final sample consisted of *N* = 984 participants, of whom 65.0% identified as female, 33.6% as male, and 0.01% as non-binary or other. The mean age was 37.4 (*SD* = 13.2) years.

#### Experimental design and stimuli

The experiment had a between-subjects design with random assignment to one of three advertisement conditions: simulation-enhancing, health-focused, or control. In the simulation-enhancing and health-focused conditions, participants viewed three advertisements for a fictitious brand of bottled water with slogans referring either to consumption and reward (i.e., “Invigorate your body with a cool splash of Aquaviva”), or to health (i.e., “Aquaviva takes care of your health”). In the control condition, participants viewed three advertisements for a fictitious insurance company. Within each condition, the order in which the three advertisements were presented was randomized and each individual advertisement was shown to participants for 30s. Participants were instructed to immerse themselves in the situation depicted in the advertisement, and the instructions were customized for each advertisement condition (see Fig. 1 and Fig. S1 in the SOM).

#### Procedure and measures

Participants were first asked to rate their current thirst level (*M* = 47.4, *SD* = 25.4) on a 0–100 Visual Analog Scale (VAS) with the anchors 0 = *not at all*, 50 = *somewhat*, 100 = *definitely*. They were then presented with the advertisements and were instructed according to the condition they were assigned to.

After viewing the advertisements, participants were presented with the Feature Listing task. They were shown the bottled water depicted in the ads and were asked to describe the bottled water using five words or short phrases: “How would you describe this drink now? Please try to fill in all 5 boxes. Type in what comes to mind spontaneously”.

After this task, participants were asked to rate the bottled water on four measures responding on a 0-100 VAS with the anchors 0 = *not at all*, 50 = *somewhat*, 100 = *very/completely*. First, they were asked to rate anticipated reward for the bottled water: “To what extent do you agree with the following statements? a) This drink would taste very nice, b) This drink would be refreshing, c) This drink would make me feel energized, d) I would really enjoy drinking this drink.” The Cronbach’s alpha of these items was ⍺ = 0.87, so they were combined into mean scores. Participants then rated the bottled water on attractiveness: “How attractive do you find this drink?”, and desire: “How much they would you like to drink this drink right now?” Next, they were asked to rate the anticipated health benefits of the bottled water: “To what extent do you agree with the following statements? a) This drink is healthy, b) This drink would help me reach my health goals, c) This drink would be good for my body in the long-term.” The Cronbach’s alpha of these items was ⍺ = 0.83, so they were combined into mean scores.

Participants were then asked their WTP for the bottled water (*M* = 0.67, *SD* = 0.30): “How much are you willing to pay for this drink?” On a slider scale, they could choose any number between £0 and £1.50 (with increments of £0.01). Next, participants were asked to imagine being offered a choice between the bottled water they saw in the advertisements and a Coca-Cola and were asked which one they would choose to drink at that moment.

Finally, participants reported their consumption frequency of bottled water (any type), tap water, and soft drinks on a 0–100 VAS with anchors 0 = *never*, 50 = *sometimes*, 100 = *very often*. Finally, we collected self-reported demographic information, including age, height and body weight, before participants were debriefed, thanked, and paid.

#### Coding of feature listing entries

The features that participants listed in the Feature Listing task were coded using a hierarchical coding scheme developed to code features of foods and drinks (see [31] and the ShinyApp that facilitated the coding: https://niklasjohannes.shinyapps.io/feature_coding/). The main features of interest were consumption and reward features and positive long-term health consequences features. The proportion of consumption and reward features was calculated by summing the proportions of sensory and action features (e.g., “sweet”, “cold”, “fizzy”), contextual features (e.g., “with salty food”, “with friends”), and immediate positive consequences (e.g., “tasty”, “thirst quenching”), and then dividing this total by the total number of features generated per participant. Similarly, the proportion of positive long-term health features (e.g., “good for you”, “healthy”) was calculated by dividing these features by the total number of features listed by the participant.

Similar to previous findings [12], participants listed an average of 4.59 (*SD* = 0.72) features. Moreover, the average proportion of consumption and reward features was 0.45 (*SD* = 0.29), and 0.10 (*SD* = 0.15) for positive long-term health consequences features. These values were similar across all three experiments in the current study.

#### Analysis plan

To test the effect of advertisement condition, we used binomial regressions for the proportion of consumption and reward features, *t*-tests (Welch’s method) for ratings of attractiveness, anticipated reward, anticipated health, and desire, and Chi-squared tests to examine the choice for bottled water versus Coca Cola. Confirmatory tests were evaluated based on one-tailed and exploratory on two-tailed tests. We adjusted our alpha levels for our confirmatory hypotheses using Bonferroni to control for multiple comparisons and multiple testing (see SOM). For the mediation analyses we used the *lavaan* package [32]. All analyses were conducted in *R* [33].

All experiments were sequentially pre-registered on the Open Science Framework where all materials, data, and analysis scripts can be accessed [34]. Hypotheses were specified prior to data collection. We clearly distinguish between confirmatory analyses to test our hypotheses and exploratory analyses. Additional results from covariate assessments and exploratory analyses can be found in the SOM, but do not bear on the main findings.

### Results

#### Confirmatory analyses

##### The effect of advertisement condition on representations in terms of consumption and reward (Hyp. 1)

**Fig. 2:**
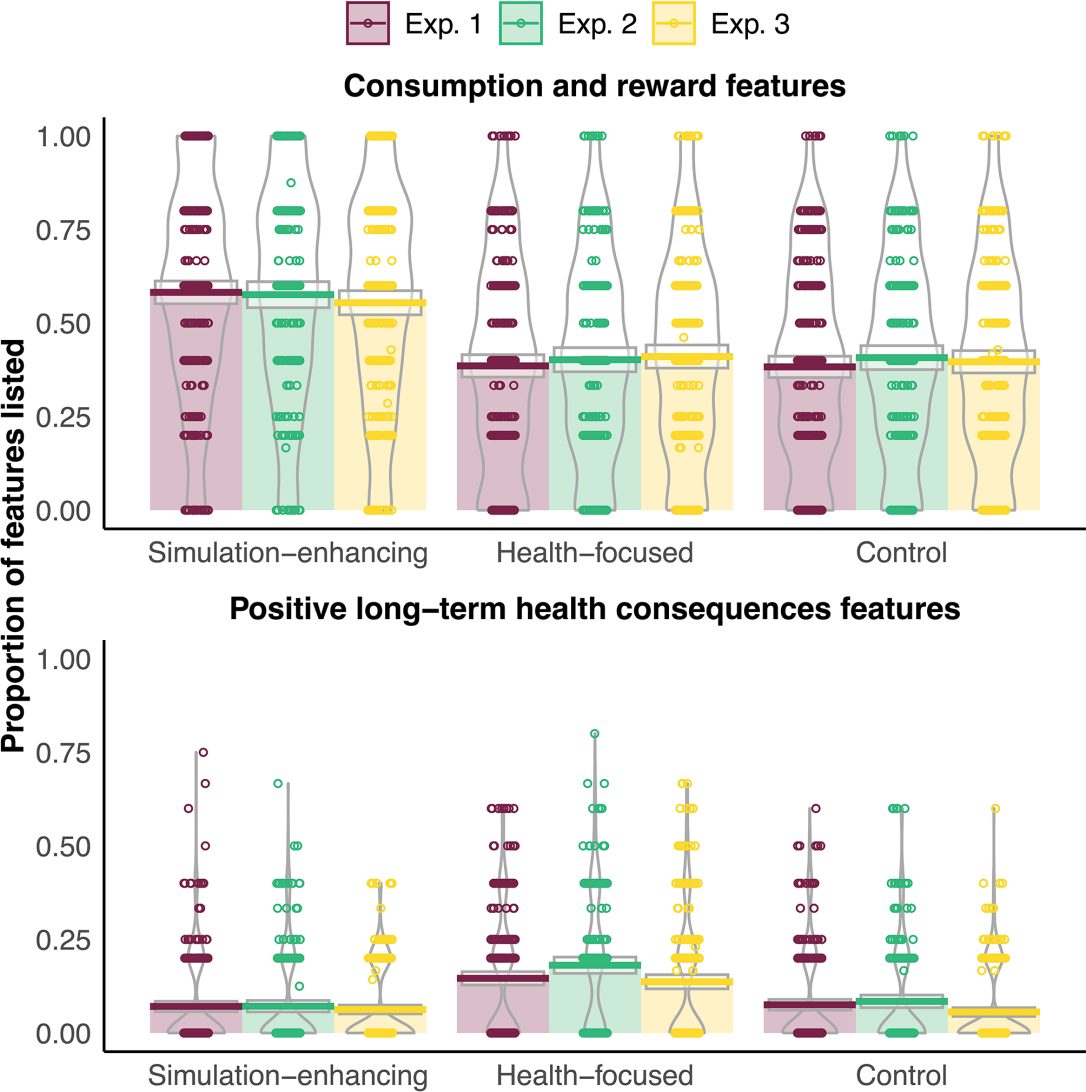
The proportion of consumption and reward features and positive long-term health consequences features listed for bottled water in the Feature Listing task across the different advertisement conditions in Exp. 1, Exp. 2, and Exp. 3. Pirate plots showing raw data as points, bars and horizontal lines indicating the means with boxes representing 95% confidence intervals, and beans showing the density

Consistent with our hypotheses, participants represented the bottled water more in terms of consumption and reward as assessed by the Feature Listing task after viewing simulation-enhancing advertisements compared to health-focused or control advertisements (see Table 1 and Fig. 2).

##### The effect of advertisement condition on anticipated reward, attractiveness, desire and choice (Hyp. 2–5)

Contrary to our hypotheses, there were no differences in ratings of attractiveness, anticipated reward, and desire as measured on the 100-point VAS, or choice for bottled water vs. Coca-Cola between participants in the different advertisement conditions after controlling for multiple comparisons (see Table 1).

**Table 1:** Means and test statistics comparing the variables of interest in the different advertisement conditions for Exp. 1–3

		Control ads	Health-focused ads	Simulation-enhancing ads	Control vs. simulation enhancing ads	Health-focused vs. simulation enhancing ads	Control vs. Health-focused ads
		*M* (*SD*)					
Consumption and reward features	Exp. 1	0.38 (0.26)	0.39 (0.28)	0.58 (0.28)	*b* = 0.81, *SE* = 0.16, *p* < .001	*b* = 0.80, *SE* = 0.16, *p* < .001	*b* = 0.01, *SE* = 0.16, *p* = .943
	Exp. 2	0.41 (0.26)	0.40 (0.27)	0.58 (0.29)	*b* = 0.68, *SE* = 0.18, *p* < .001	*b* = 0.70, *SE* = 0.18, *p* < .001	*b* = -0.02, *SE* = 0.18, *p* = .898
	Exp. 3	0.40 (0.27)	0.41 (0.27)	0.55 (0.29)	*b* = 0.63, *SE* = 0.17, *p* < .001	*b* = 0.58, *SE* = 0.16, *p* < .001	*b* = 0.06, *SE* = 0.17, *p* = .727
Positive long-term health consequences features	Exp. 1	0.08 (0.13)	0.15 (0.17)	0.07 (0.13)	*b* = -0.07, *SE* = 0.30, *p* = .827	*b* = -0.81, *SE* = 0.27, *p* = .002	*b* = 0.74, *SE* = 0.26, *p* = .005
	Exp. 2	0.08 (0.14)	0.18 (0.18)	0.07 (0.13)	*b* = -0.18, *SE* = 0.33, *p* = .579	*b* = -1.05, *SE* = 0.29, *p* < .001	*b* = 0.87, *SE* = 0.27, *p* = .002
	Exp. 3	0.06 (0.10)	0.14 (0.17)	0.06 (0.10)	*b* = 0.12, *SE* = 0.34, *p* = .364	*b* = -0.86, *SE* = 0.29, *p* = .002	*b* = 0.98, *SE* = 0.30, *p* = .001
Attractiveness	Exp. 1	49.5 (28.3)	47.8 (27.5)	49.1 (26.4)	*t* = -0.20, *df* = 651, *p* = .578	*t* = 0.62, *df* = 653, *p* = .268	*t* = -0.80, *df* = 653, *p* = .430
Anticipated reward	Exp. 1	66.9 (21.3)	67.9 (20.1)	69.8 (20.4)	*t* = 1.83, *df* = 653, *p* = .034	*t* = 1.20, *df* = 654, *p* = .116	*t* = 0.67, *df* = 652, *p* = .504
	Exp. 2	67.6 (21.7)	68.7 (19.9)	70.0 (20.8)	*t* = -1.25, *df* = 522, *p* = .210	*t* = -0.73, *df* = 521, *p* = .464	*t* = -0.56, *df* = 515, *p* = .576
	Exp. 3	67.1 (19.9)	68.5 (19.3)	69.2 (21.2)	*t* = -1.26, *df* = 600, *p* = .209	*t* = -0.45, *df* = 597, *p* = .651	*t* = -0.85, *df* = 602, *p* = .395
Anticipated health benefits	Exp. 1	86.5 (16.3)	80.2 (18.8)	82.5 (18.8)	*t* = -2.86, *df* = 641, *p* = .004	*t* = 1.62, *df* = 654, *p* = .106	*t* = -4.58, *df* = 641, *p* < .001
	Exp. 2	86.3 (17.7)	81.0 (19.3)	84.5 (16.7)	*t* = 1.20, *df* = 521, *p* = .232	*t* = -2.20, *df* = 507, *p* = .028	*t* = 3.24, *df* = 513, *p* = .001
	Exp. 3	84.4 (17.4)	82.3 (18.2)	84.2 (17.5)	*t* = 0.12, *df* = 602, *p* = .906	*t* = -1.32, *df* = 602, *p* = .186	*t* = 1.45, *df* = 602, *p* = .149
Desire	Exp. 1	56.6 (29.6)	54.1 (30.2)	56.3 (29.3)	*t* = -0.15, *df* = 654, *p* = .558	*t* = 0.95, *df* = 654, *p* = .172	*t* = -1.09, *df* = 654, *p* = .278
	Exp. 2	57.6 (29.9)	57.1 (28.6)	58.5 (28.8)	*t* = -0.33, *df* = 523, *p* = .743	*t* = -0.56, *df* = 522, *p* = .573	*t* = 0.22, *df* = 517, *p* = .823
	Exp. 3	61.8 (28.8)	61.9 (28.0)	62.8 (29.0)	*t* = -0.40, *df* = 602, *p* = .687	*t* = -0.36, *df* = 602, *p* = .717	*t* = -0.05, *df* = 602, *p* = .963
Willingness To Pay	Exp. 1	0.66 (0.30)	0.66 (0.30)	0.69 (0.30)	*t* = 1.04, *df* = 654, *p* = .299	*t* = 1.01, *df* = 654, *p* = .312	*t* = 0.02, *df* = 654, *p* = .494
	Exp. 2	0.69 (0.32)	0.73 (0.31)	0.73 (0.32)	*t* = -1.68, *df* = 524, *p* = .094	*t* = -0.24, *df* = 522, *p* = .811	*t* = -1.45, *df* = 518, *p* = .147
	Exp. 3	0.68 (0.32)	0.67 (0.35)	0.71 (0.34)	*t* = -1.31, *df* = 600, *p* = .192	*t* = -1.42, *df* = 602, *p* = .155	*t* = 0.19, *df* = 597, *p* = .851
		Binary choices					
Choice	Exp. 1	247 water, 81 Coca-Cola	250 water, 78 Coca-Cola	247 water, 81 Coca-Cola	χ^2^ = 0.00, *df* = 1, *p* = 1.00	χ^2^ = 0.03, *df* = 1, *p* = .855	χ^2^ = 0.03, *df* = 1, *p* = .855
Self-reported drinking simulations	Exp. 2	63.1 (26.4)	60.6 (26.0)	63.6 (27.5)	*t* = -0.17, *df* = 524, *p* = .862	*t* = -1.28, *df* = 521, *p* = .201	*t* = 1.13, *df* = 518, *p* = .261
	Exp. 3	62.9 (25.7)	63.4 (25.5)	63.0 (25.5)	*t* = -0.05, *df* = 602, *p* = .962	*t* = 0.21, *df* = 603, *p* = .833	*t* = -0.26, *df* = 603, *p* = .796

*Note.* Test statistics are provided for all contrasts for completeness, the relevant ones assessing our hypotheses or exploratory analyses are mentioned in the text

##### Mediation through perceptions of anticipated reward (Hyp. 3b and 4b)

Although there was no difference in attractiveness and desire ratings of bottled water after viewing simulation-enhancing advertisements compared to control or health-focused advertisements, we conducted the pre-registered mediation analyses in accordance with current practices which allows this in the absence of a direct effect [35–37]. Contrary to our hypotheses, results from these analyses revealed no significant indirect effects of advertisements through anticipated reward ratings on either attractiveness (simulation-enhancing vs. health-focused: *b* = 1.58, *p* = .231; simulation-enhancing vs. control: *b* = 2.69, *p* = .068), or desire (simulation-enhancing vs. health-focused: *b* = 1.94, *p* = .231; simulation-enhancing vs. control: *b* = 2.98, *p* = .068).

#### Exploratory analyses

##### The effect of advertisement condition on willingness to pay

There was no difference in WTP for the bottled water between participants in the different advertisement conditions (see Table 1).

##### The effect of advertisement condition on representations in terms of positive long-term health consequences

Analyses of positive long-term health consequences features as measured by the Feature Listing task showed that after viewing health-focused compared to simulation-enhancing and control advertisements, participants represented the bottled water more in terms of positive long-term health consequences (see Table 1 and Fig. 2).

##### The effect of advertisement condition on anticipated health benefits

There was no difference in ratings of anticipated health benefits as measured on the 100-point VAS for the bottled water after viewing health-focused advertisements compared to simulation-enhancing advertisements. Surprisingly, however, compared to control advertisements, participants reported fewer anticipated health benefits after viewing health-focused advertisements (see Table 1).

##### Mediation through shifts in cognitive representations

**Fig. 3:**
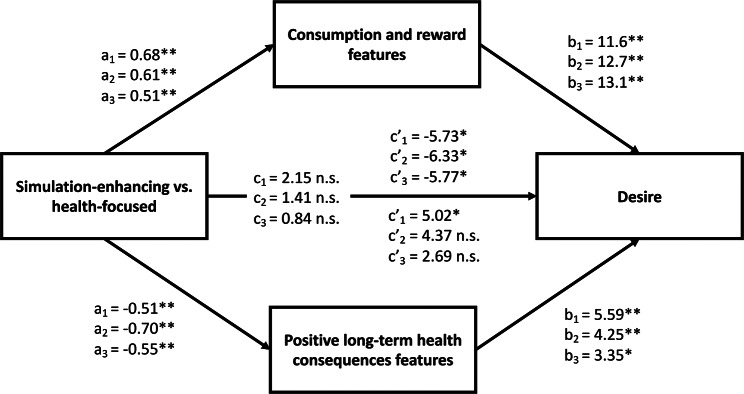
Pathways showing how advertisements increased desire through a change in cognitive representations as assessed by the Feature Listing task. Simulation-enhancing advertisements increased desire through an increase in the proportion of consumption and reward features, and health-focused advertisements increased desire through an increase in positive long-term health consequences features. See Table 2 for the coefficients of the indirect effects. *Note*. Health-focused was coded as the reference category, hence a positive *a* coefficient indicates an increase due to the simulation-enhancing advertisement, and a negative *a* coefficient indicates an increase due to the health-focused advertisement. Results were similar when desire was replaced by WTP and when the simulation-enhancing and health-focused advertisements were compared with the control advertisements (see Table 2 and Fig. S4 in the SOM). **p < .001, *p < .05, n.s. not significant

We explored whether the proportions of consumption and reward features and positive long-term health consequences features played a role in the indirect effects of advertisement condition on desire and WTP, in separate mediation models. While there were no direct effects of advertisements on these variables, indirect pathways were significant, as we describe next (see Table 2; Fig. 3 and Fig. S4 in the SOM).

**Table 2:** Indirect effects suggesting mediation through representations of water through consumption and reward and through positive long-term health consequences, across the three experiments

**Mediation through consumption and reward features**										
		**Experiment 1**			**Experiment 2**			**Experiment 3**		
		*b* (*SE*)	95% CI		*b* (*SE*)	95% CI		*b* (*SE*)	95% CI	
			Lower	Upper		Lower	Upper		Lower	Upper
Health-focused vs. simulation-enhancing	Desire	7.89 (1.16)	5.67	10.2	7.74 (1.29)	5.22	10.27	6.61 (1.17)	4.32	8.91
	WTP	0.07 (0.01)	0.05	0.09	0.07 (0.01)	0.05	0.10	0.06 (0.01)	0.04	0.08
Control vs. simulation-enhancing	Desire	8.00 (1.16)	5.71	10.3	7.60 (1.30)	5.06	10.14	7.33 (1.20)	4.97	9.69
	WTP	0.06 (0.01)	0.04	0.08	0.07 (0.01)	0.04	0.09	0.06 (0.01)	0.04	0.08
**Mediation through positive long-term health consequences features**										
		**Experiment 1**			**Experiment 2**			**Experiment 3**		
		*b* (*SE*)	95% CI		*b* (*SE*)	95% CI		*b* (*SE*)	95% CI	
			Lower	Upper		Lower	Upper		Lower	Upper
Health-focused vs. simulation-enhancing	Desire	-2.87 (0.73)	-4.30	-1.44	-2.95 (0.95)	-4.81	-1.09	-1.85 (0.68)*	-3.17	-0.53
	WTP	-0.03 (0.01)	-0.04	-0.01	-0.03 (0.01)	-0.05	-0.01	-0.04 (0.01)	-0.05	-0.02
Control vs. health-focused	Desire	2,99 (0.73)	1.56	4.42	4.04 (0.95)	2.17	5.91	1.82 (0.71)	0.42	3.22
	WTP	0.02 (0.01)	0.01	0.04	0.06 (0.01)	0.03	0.08	0.04 (0.01)	0.02	0.06

*Note*. Standardized coefficients are provided. The first condition mentioned in the contrast was coded as the reference category.

* *p* < .01, all other effects were significant at *p* < .001

##### Mediation through representations in terms of consumption and reward

There were significant indirect effects of simulation-enhancing advertisements on desire and WTP, through the proportion of consumption and reward features, compared to health-focused and control advertisements. In other words, simulation-enhancing advertisements increased consumption and reward features listed, which in turn increased desire and WTP for the bottled water.

##### Mediation through representations in terms of positive long-term health consequences

There were significant indirect effects linking health-focused advertisements to more desire and WTP, through the proportion of positive long-term health consequences features, compared to simulation-enhancing and control advertisements. In other words, health-focused advertisements increased representations in terms of positive long-term health consequences features listed, which in turn increased desire and WTP for the bottled water.

### Discussion

In this experiment, we found that participants represented a bottled water more in terms of consumption and reward features after mentally immersing themselves in simulation-enhancing advertisements compared to health-focused or control advertisements. In addition, bottled water was represented more in terms of health features following the viewing of health-focused compared to simulation-enhancing or control advertisements. This suggests that in combination with our immersive instructions, which may have enhanced the effects of the advertisements, the advertisements shifted participants’ representations of water towards simulations of consuming and enjoying it, or towards its health implications.

Contrary to our expectations, there were no significant effects of advertisements on desire, nor indirect effects through ratings of anticipated reward. However, we did find significant indirect effects showing that simulation-enhancing advertisements increased desire and WTP through an increase in consumption and reward representations. Similarly, health-focused advertisements increased attractiveness, desire, and WTP through increased representations of health consequences. These findings suggest that the advertisements and immersive instructions to imagine being in the depicted situation prompted participants to simulate the pleasurable experience (in the simulation-enhancing condition) or the positive long-term health consequences of drinking water (in the health-focused condition). While this did not affect explicit perceptions of reward or health, these simulations were detected by our Feature Listing task which assessed cognitive representations, indicating a shift in representations in response to the advertisements. In addition, representations in terms of consumption and reward as well as positive long-term health consequences increased motivation to consume water, as assessed by ratings of desire and WTP.

While indirect pathways were significant, there were no significant main effects (as tested by Hyp. 1) or total effects of advertisement conditions on motivation for bottled water. Current recommendations for mediation analyses propose that indirect pathways can be significant and meaningful to interpret in the absence of these effects [37, 38]. These patterns can occur, for example, when the effect of the mediator on the dependent variable is stronger than the direct effect. In this experiment, the effect of condition on desire may have been reduced by the influence of completing the Feature Listing task (mediator) on subsequently reported desire. This may not have occurred if desire had been measured immediately after viewing the advertisements. Alternatively, the effect may be too small to be detected with our sample or may not be strong enough to directly influence motivation in the presence of competing information such as thirst, water consumption habits, water liking, or general expectations of how much a bottle of water should cost.

Furthermore, the absence of a total effect may be attributed to the direct effect (main effect when controlling for the mediator) and indirect effect having opposing signs (i.e., suppression effect; [36]). It is likely that our two mediators acted as suppressors of the other’s pathway. For example, we found two opposing pathways through which simulation-enhancing and health-focused advertisements influence desire: through representations of consumption and reward and through health perceptions. In each of our mediation models, the nonsignificant direct effect of advertisement condition on motivation was reduced to such an extent that it became significant, albeit in the opposite direction, suggesting that the presence of these two significant indirect pathways indeed competed with each other [37]. In other words, the findings from this exploratory test suggest that although desire and WTP were similar across conditions, simulation-enhancing advertisements indirectly influenced desire and WTP by increasing participants’ representations of consumption and reward, and health-focused advertisements indirectly influenced desire and WTP by increasing participants’ representations of water as healthy.

An alternative explanation for the effects of the advertisements and immersive instructions on our Feature Listing task might be that participants simply copied the words they read in the advertisements. However, a closer examination of the words listed suggests that this is not the case: Participants used a wide variety of words to describe the bottled water, ranging from 376 to 419 unique features across conditions. Moreover, when we looked at the overlap between the features listed in the conditions, we found that approximately 28% of the features were common to all three conditions, 29% of the features overlapped between sets of two of the three conditions, leaving 43% of the features unique to the specific conditions (see the SOM for more details). These observations suggest that participants relied on language reflecting their idiosyncratic representations of the bottled water rather than merely copying the words they saw in the advertisements.

The results of Exp. 1 suggest that desire for bottled water is predicted by representations in terms of consumption and enjoyment after deep processing of simulation-enhancing advertisements, and by representations of long-term health benefits after deep processing of health-focused advertisements. One possible explanation for why emphasizing the health benefits of water may increase desire for water is that people may experience positive affect when imagining the positive long-term consequences of drinking water for their health. Previous research suggests that health information may increase perceptions of tastiness of foods and drinks, particularly if the product is neutral and if individuals attach importance to health-related aspects of eating [39, 40]. This may also explain why ratings of anticipated health benefits were lower for the bottled water after being immersed in health-focused ads compared to control ads: While health is an inherent characteristic of water, deep processing of the health benefits of water may have triggered simulations of reward. Alternatively, the health-focused ads used in this experiment may have been appealing because they contained both health information and images that may have induced simulations of consumption and reward (e.g., an image of two people seemingly enjoying drinking the water).

To address this latter point, we replicated Exp. 1, by adjusting the advertisements in the health-focused condition to emphasize only the health benefits of water, without also inducing consumption simulations. This was done by (1) changing the images to match the text in the advertisements and emphasizing the health benefits of water, hence differentiating them more from the images in the simulation-enhancing advertisements, and by (2) removing all references to the purity of water in the health-focused text and replacing them with health-related words.

In addition, in Exp. 2, for a more robust test of the direct effect, we assessed desire and WTP before assessing ratings of anticipated reward and health. Moreover, we included a measure assessing self-reported drinking simulations. This allowed us to assess whether imagining the positive long-term health consequences of water increases motivation in a similar way to when imagining water in terms of consumption and enjoyment, namely through cognitive representations that trigger simulations of drinking water.

## Experiment 2

In Exp. 2, we tested the hypotheses that simulation-enhancing advertisements would increase representations in terms of consumption and reward features (Hyp. 1a), and that these features would mediate the effect of simulation-enhancing advertisements on desire and WTP compared to health-focused or control advertisements (mediation; Hyp. 1b and 1c). In addition, we predicted that health-focused advertisements would increase representations in terms of positive long-term health consequences features (Hyp. 2a), and that these features would mediate the effect of health-focused advertisements on desire and WTP compared to simulation-enhancing and control advertisements (mediation; Hyp. 2b and 2c). In addition, we predicted that the indirect pathway through consumption and reward features would be stronger than that through positive long-term health consequences features (Hyp. 3). Finally, we explored whether the indirect effects of advertisement on desire and WTP through the proportion of positive long-term health consequences features could be explained by increased self-reported drinking simulations (serial mediation).

### Method

#### Participants

Power analyses suggested a minimum sample size of *N* = 765 (see SOM for more details). Participants were invited in the same way and excluded according to the same criteria as in Exp. 1. We collected data from *N* = 786 participants, excluding one participant who took more than four times the median time to complete the study. The final sample consisted of *N* = 785 participants of whom 65% identified as female, 34% as male, and 1% as non-binary or other. The mean age was 40.0 (*SD* = 13.7) years.

#### Experimental design and stimuli

Like Exp.1, the experiment had a between-subjects design with random assignment to three advertisement conditions: simulation-enhancing, health-focused, or control. The advertisements used in the simulation-enhancing and control conditions were the same as in Exp.1. For the health-focused advertisements, we removed all references to purity from the slogans by replacing “purest” with “healthiest”, and purifying” with “nourishing”. In addition, we conducted a pilot study to select new images for the health-focused advertisements.

##### Piloting the advertisements

We designed a pilot study to select new images for the health-focused advertisements that would be similar to those in the simulation-enhancing advertisements in terms of (1) valence, (2) the extent to which people can imagine being in the situation shown in the advertisement, and (3) how well the slogan fits the images in the advertisement. A sample of *N* = 200 (*M*_age_ = 41.4, *SD*_age_ = 13.2, 69% female) participants were asked to rate the images on these three characteristics. We piloted a total of 24 new health-focused advertisements (8 new images paired with each of the 3 slogans) and selected those that best matched the simulation-enhancing ads using one-to-one propensity score matching (see the SOM for more details on this pilot and Fig. S5 in SOM for the final selection of the health-focused advertisements).

#### Procedure and measures

The procedure and measures were almost identical as to those in Exp. 1. First, participants were asked to rate their thirst (*M* = 47.1, *SD* = 25.7). They were then shown the same instructions as in Exp. 1, where they were asked to take a close look at each advertisement and try to immerse themselves in it, again customized for each advertisement condition. They then completed the Feature Listing task, and rated the bottled water on desire and WTP. Next, participants were asked to rate their experience of drinking simulations (⍺ = 0.89) when seeing the bottled water with the following items: “When I see this drink… a) I imagine drinking it, b) I imagine what it would taste like, c) I imagine what it would feel like in my mouth.” Responses were recorded on a 0-100 VAS with the anchors 0 = *not at all*, 100 = *very/completely*. Participants then rated the anticipated reward (⍺ = 0.88) and anticipated health benefits of water (⍺ = 0.86), their drinks consumption frequency, and provided demographic information, before being debriefed, thanked, and paid.

We removed the question about the attractiveness of the bottled water given that results for attractiveness and desire were very similar and that ratings on these items were highly correlated in Exp. 1 (*r* = .646, *p* < .001). We also removed the choice task since participants were much more likely to select the bottled water over the Coca-Cola across all conditions in Exp. 1.

#### Analysis plan

The analysis plan was identical to that in Exp. 1. In addition, to compare the relative strength of the two indirect effects, we carried out a Likelihood Ratio test [41].

### Results

#### The effect of advertisement condition on consumption and reward features (Hyp. 1a)

Consistent with our hypothesis and findings in Exp. 1, participants represented the bottled water more in terms of consumption and reward as assessed by the Feature Listing task after viewing simulation-enhancing advertisements compared to health-focused advertisements, or control advertisements (see Table 1; Fig. 2).

##### Mediation through representations of consumption and reward (hyp. 1b and 1c)

There were significant indirect effects linking simulation-enhancing advertisements to more desire and WTP through the proportion of consumption and reward features, compared to health-focused and control advertisements (see Table 2; Fig. 3, and Fig. S4 in the SOM).

#### The effect of advertisement condition on positive long-term health consequences features (Hyp. 2a)

Consistent with our hypothesis and findings in Exp. 1, participants represented the bottled water more in terms of positive long-term health consequences as assessed by the Feature Listing task after viewing health-focused advertisements compared to simulation-enhancing advertisements, or control advertisements (see Table 1 and Fig. 2).

##### Mediation through representations of positive long-term health consequences (Hyp. 2b and 2c)

There were significant indirect effects linking health-focused advertisements to more desire and WTP through the proportion of positive long-term health consequence features, compared to simulation-enhancing and control advertisements (see Table 2; Fig. 3, and Fig. S4 in the SOM).

#### Comparing the relative strength of the indirect effects (Hyp. 3)

We used a Likelihood Ratio test to compare the relative strength of the two indirect effects through consumption and reward features and positive long-term health consequences features. This analysis indicated that models including both indirect effects from advertisement on desire and WTP were significantly different from models that constrained these effects to be equal in magnitude, χ^2^ = 73.0, *df* = 1, *p* < .001 for desire, and χ^2^ = 68.0, *df* = 1, *p* < .001 for WTP. Thus, we can conclude that the pathways through representations of consumption and reward features were significantly stronger than those through positive long-term health consequences features (see Fig. 3).

#### Exploring serial mediation through self-reported drinking simulations

**Fig. 4:**
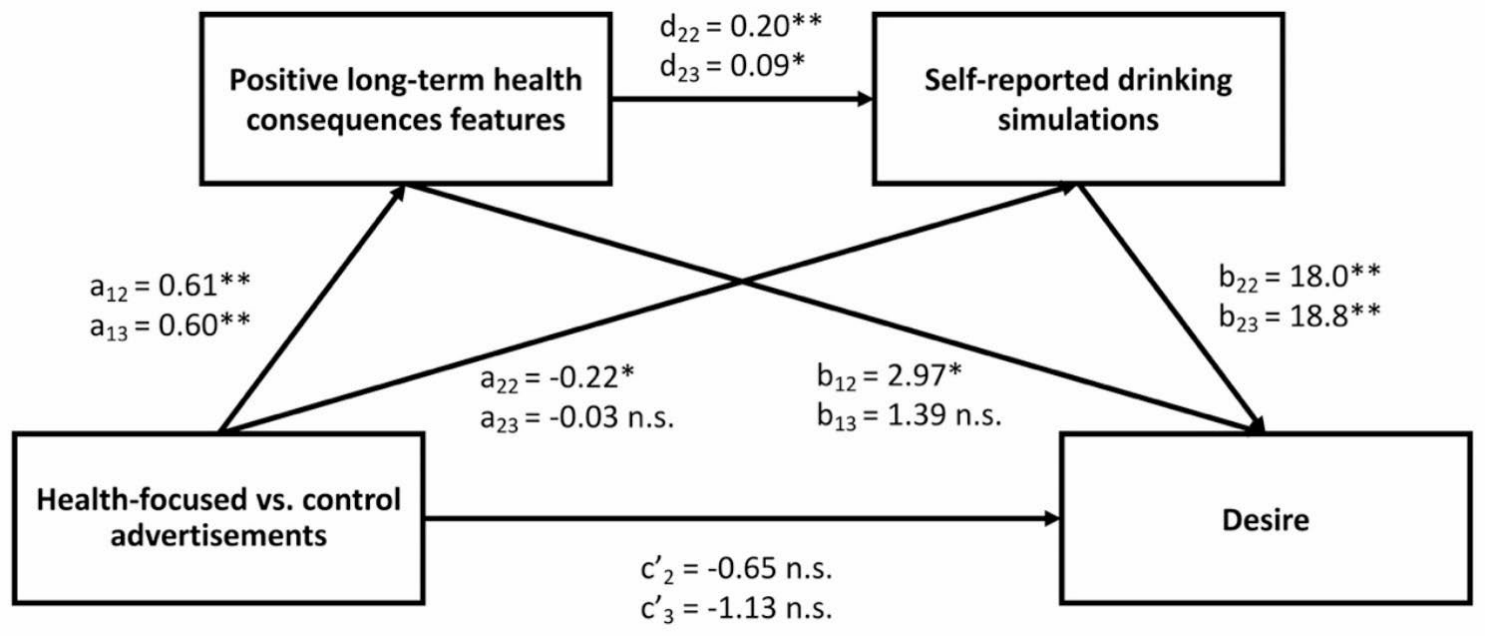
Serial mediation linking health-focused (vs. control) advertisements to desire and WTP through representations of positive long-term health consequences as assessed by the Feature Listing task and self-reported drinking simulations. See text for the coefficients of the indirect effects. *Note*. Health-focused was coded as the reference category. The second number in the subscript refers to the corresponding experiment number (Exp. 2 and 3). Results were similar when desire was replaced by WTP (see SOM). **p < .001, *p < .05, n.s. not significant

Finally, we explored whether the effects of advertisement conditions on desire and WTP through the proportion of positive long-term health consequence features (as assessed by the Feature Listing task) could be explained by increased self-reported drinking simulations. Serial mediations with 5000 bootstrapped samples confirmed significant indirect effects through the proportion of positive long-term health consequences features and self-reported drinking simulations on desire, *b =* 2.22, *SE* = 0.57, *p* < .001, 95% CI [1.10, 3.35] and WTP, *b =* 0.02, *SE* = 0.01, *p* < .001, 95% CI [0.01, 0.02]. (See Fig. 4 and Fig. S7 in the SOM which also presents additional mediation analyses to establish the robustness of the indirect effects [42]).

These results suggest that increased representations of positive long-term health consequences features after viewing health-focused advertisements compared to control advertisements increased desire and WTP for bottled water due to increased simulations of the sensory experience of drinking it.

### Discussion

The results of Exp. 2 replicate those of Exp. 1: Participants who immersed themselves in simulation-enhancing advertisements represented the bottled water more in terms of consumption and reward, and those who immersed themselves in health-focused advertisements represented the bottled water more in terms of positive long-term health consequences. Again, advertisement conditions had no direct effect on motivation, but significant indirect effects showed that simulation-enhancing advertisements increased desire and WTP through representations of consumption and reward, and health-focused advertisements increased desire and WTP through representations of water in terms of positive long-term health consequences, as assessed by a Feature Listing task. The effects through representations of consumption and reward were stronger than through positive long-term health consequences, suggesting that targeting these representations in advertisements may be more effective in increasing motivation for bottled water. Lastly, exploratory analyses indicated that thinking about the positive long-term health consequences of water increased desire for water through simulations of consuming water.

In Exp. 1 and 2, participants were asked to immerse themselves in the situations shown in the advertisements for 30s and were given detailed instructions on which aspects of the ads to focus on. However, this may not be the way that people typically process advertisements in real-life situations. To address this limitation, in Exp. 3, we replicated the procedure in a more naturalistic way by merely asking participants to view each ad for 10s. This is closer to how consumers may process advertisements in everyday situations outside of the laboratory. Previous research has also shown that 10s is the duration at which the effect of exposure on advertisement recognition and recall starts to diminish [43].

## Experiment 3

In Exp. 3, we tested the same hypotheses as in Exp. 2. In addition, we now formally predicted the indirect effects of health-focused vs. control advertisement on desire and WTP via representations of positive long-term health consequences and self-reported drinking simulations (serial mediation; Hyp 4). For a stronger test of this serial mediation, we now assessed self-reported drinking simulations before assessing drinking motivation.

### Method

#### Participants

Power analyses suggested a minimum sample size of *N* = 900 (see SOM for more details). Participants were invited in the same way and excluded according to the same criteria as in Exp. 1 and 2. We collected data from *N* = 917 participants, excluding participants who took more than four times the median time to complete the study (*n* = 1) and those with incomplete responses (*n* = 9). The final sample consisted of *N* = 907 participants of whom 72% identified as female, 27% as male, and 1% as non-binary or other. The mean age was 38.4 (*SD* = 12.8) years.

#### Experimental design and stimuli

As in Exp.1 and 2, the study had a between-subjects design with random assignment to three advertisement conditions: simulation-enhancing, health-focused, or control. The advertisements used in the simulation-enhancing and health-focused conditions were the same as those used in Exp. 2. For the control condition, three new advertisements were created featuring the bottled water in a neutral outdoor environment with no references to reward or health aspects (see Fig. S8 in the SOM). In addition, all participants saw three filler advertisements for an insurance company.

#### Procedure and measures

The procedure and measures were almost identical as to those in Exp. 1 and 2. First, participants were asked to rate their thirst (*M* = 50.2, *SD* = 24.7). Then, participants were asked to simply view the advertisements for 10s. Next, they completed the Feature Listing task and rated their experience of drinking simulations (⍺ = 0.88), desire for the bottled water, and WTP. Finally, participants rated the anticipated reward (⍺ = 0.88) and anticipated health benefits of the water (⍺ = 0.86), their drink consumption frequency, and provided demographic information, before being debriefed, thanked, and paid.

### Results

#### The effect of advertisement condition on consumption and reward features (Hyp. 1a)

Consistent with our hypothesis and findings in Exp. 1 and 2, participants represented the bottled water more in terms of consumption and reward features after viewing simulation-enhancing advertisements compared to health-focused or control advertisements (see Table 1; Fig. 2).

##### Mediation through representations of consumption and reward (Hyp. 1b and 1c)

Again, as predicted, there were significant indirect effects linking simulation-enhancing advertisements to more desire and WTP through the proportion of consumption and reward features, compared to health-focused and control advertisements.

#### The effect of advertisement condition on positive long-term health consequences (Hyp. 2a)

Consistent with our hypothesis and findings in Exp. 1 and 2, participants represented the bottled water more in terms of positive long-term health consequences features after viewing health-focused advertisements compared to simulation-enhancing and control advertisements (see Table 1 and Fig. 2).

##### Mediation through representations of positive long-term health consequences (Hyp. 2b and Hyp. 2c)

Again, as predicted, there were significant indirect effects linking health-focused advertisements to more desire and WTP through the proportion of positive long-term health consequences features, compared to simulation-enhancing and control advertisements.

#### Comparing the relative strength of the indirect effects (Hyp. 3)

A Likelihood-Ratio test indicated that models including both indirect effects from advertisement on desire and WTP were significantly different from models that constrained these effects to be equal in magnitude, χ^2^ = 46.5, *df* = 1, *p* < .001 for desire, and χ^2^ = 56.5, *df* = 1, *p* < .001 for WTP. Thus, as in Exp. 2 and as hypothesized, the pathways through consumption and reward features were significantly stronger than those through positive long-term health consequences features (see Fig. 3).

#### Serial mediation through self-reported drinking simulations (Hyp. 4)

Finally, consistent with our hypothesis and as in Exp. 2, serial mediations with 5000 bootstrapped samples confirmed significant indirect effects through the proportion of positive long-term health consequences features and self-reported drinking simulations on desire, *b =* 0.98, *SE* = 0.47, *p* = .035, 95% CI [0.07, 1.89], and WTP, *b =* 0.01, *SE* = 0.01, *p* = .037, 95% CI [0.00, 0.01]. While there was no direct effect of advertisement on desire (c’_3_ = -1.13), the health-focused advertisement compared to the control advertisement increased the number of positive long-term health consequences features listed (a_13_ = 0.60), these in turn increased self-reported drinking simulations (d_23_ = 0.09) which predicted more desire (b_23_ = 18.8) and WTP (b_23_ = 0.13) for the bottled water. (See Fig. 4 and Fig.S7 in the SOM where robustness checks can be found).

### Discussion

The results of Exp. 3 replicate those of Exp. 1 and 2 using a more naturalistic method, where participants simply viewed advertisements for 10s, rather than being instructed to immerse themselves in the ads for 30s with specific instructions. Again, even with this less demanding procedure, participants represented bottled water more in terms of consumption and reward after viewing simulation-enhancing advertisements, and more in terms of positive long-term health consequences after seeing health-focused advertisements. Again, significant indirect effects showed that simulation-enhancing advertisements increased desire and WTP through the proportion of consumption and reward features, and health-focused advertisements through the proportion of positive long-term health consequences features. Again, however, we did not find any direct effects of the advertisements on desire and WTP. Critically, the effects through representations of consumption and reward were again stronger than through positive long-term health consequences. Finally, Exp. 3 replicated the finding of Exp. 2, suggesting that thinking about the positive long-term health consequences of water drinking may influence desire for water through self-reported drinking simulations.

## General discussion

In a series of three pre-registered, well-powered experiments, we found that advertisements emphasizing the rewarding or health aspects of bottled water increased desire and WTP through a shift in cognitive representations. Participants who immersed themselves in advertisements emphasizing the sensory and consumption aspects of water represented that water more in terms of consumption and reward, which in turn predicted desire and WTP for that water. Similarly, participants who immersed themselves in advertisements of a bottled water emphasizing its positive impact on health and longevity, represented this water more in terms of positive long-term health consequences, which subsequently predicted desire and WTP. While total effects were absent, we found consistent evidence of significant indirect effects across three experiments, suggesting the robustness of these findings [44]. Comparing the strength of these two indirect pathways, we found that the pathways through consumption and reward were stronger than the pathways through health. In addition, our findings suggest that health perceptions affected motivation for water through simulations of drinking water, underscoring the critical role of simulated consumption experiences in increasing desire for water.

## Theoretical and practical implications

Contrary to our hypotheses, the advertisements did not exert a direct effect on motivation for bottled water. Critically, however, they consistently induced a shift in participants’ representations of a fictitious bottled water, which in turn affected their motivation. These findings are consistent with the predictions by the grounded cognition theory of desire and motivated behavior, which describes motivation as guided by simulations of previously encountered experiences [14]. Merely by emphasizing properties inherent in bottled water (such as its refreshing nature and its health benefits), participants’ representations of the bottled water changed, at least temporarily, as indicated in their descriptions of the water during the Feature Listing task.

It could be argued that our manipulation merely primed participants with certain features of water, or in other words, increased the accessibility of consumption and reward-related information, or of health-related information, compared to the control conditions [45]. While most of the results are consistent with such a priming account, we propose that our findings indicate that participants simulated drinking and enjoying the water in response to the simulation-enhancing advertisements in particular. This is evident from the Feature Listing findings, as participants listed a variety of features that had not been mentioned in the advertisements, suggesting that they simulated actually drinking the water when asked to describe it, rather than just listing words that had been made accessible by the slogans in the advertisement.

In addition, the serial mediation analyses indicated that although health-focused advertisements increased the number of health-related words used to describe the water, their influence on desire was mediated by self-reported drinking simulations. This indirect effect suggests that our findings go beyond mere information recall, but that the advertisements indeed made participants simulate or re-experience drinking the water. In sum, our results do not conclusively establish the processes by which advertisements influence drinking motivation, but they do suggest that it is through a change in representations rather than a mere priming effect. This is also consistent with previous research showing that sensory-enhanced food advertisements increased sensory thoughts, which in turn affected taste perceptions [19]. Notably, this effect was only observed when cognitive resources were available, again suggesting that more than priming is involved.

A key question that remains unanswered is whether the effect of the ads is conditional on participants’ pre-existing representations of water. For example, the advertisements may only increase representations of consumption and reward for people who already have pre-existing positive associations with water, which would be consistent with previous findings [46]. Although we did not find moderating effects of habitual water or SSB consumption frequency or any demographic variables such as gender (see the SOM), future research may examine whether the effect of advertisements on cognitive representations of water depends on people’s pre-existing representations, whether there are other important individual differences in their effects, or whether they can be expected to work equally well across consumers.

Nevertheless, overall, we found that advertisements shifted representations and that these can be effective for increasing motivation for water. Both the effects of these representations on motivation and the indirect effects of advertisements on motivation were stronger when sensory and rewarding aspects of water were emphasized than when health was emphasized. Consistent with this, previous research [12] has shown that people tend to represent water more in terms of sensory and rewarding aspects rather than long-term health benefits, and that these representations predict desire and intake. Not liking its taste is a barrier to water consumption, while perceiving water as tasty is associated with increased and more effortless consumption [16, 47]. In addition, people report enjoying water because it makes them feel good, happy, and helps them to stay alert [16]. Overall, feeling good in the moment appears to be a key determinant of potentially habitual behaviors, and may be the key to generating health behavior change [48, 49]. Thus, in contrast to current advertising that emphasizes the purity or origin of water [50], and in contrast to interventions that mainly focus on improving knowledge about water’s positive long-term health consequences (for a systematic review, see [51]), interventions should rather emphasize the short-term rewards associated with drinking water. In line with the grounded cognition approach, healthy drinking interventions should activate positive representations of a desired behavior at moments when people need to make a beverage choice [52]. These positive representations can take many forms, such as emphasizing the sensory pleasure, the drinking enjoyment, feeling physically well after drinking, or even the approval of the behavior by others, as long as the perceived reward is immediate and is congruent with the product [14, 52].

Our results suggest that this can be achieved with simple advertisements including images and words presented for a short period of time (10s was sufficient in Exp. 3). Indeed, while Exp. 1 and 2 included deliberate and specific immersive instructions with the advertisements, that might have enhanced the ad effects, Exp. 3 had the same effects under more naturalistic viewing conditions. Thus, this approach could easily be applied in a large-scale intervention targeting the general public, for instance, in media such as television or billboards, preferably in specific consumption settings such as vending machines or at take-out counters. Given that previous research has shown spillover effects from auditory input to taste perceptions of food and beverages [53], one could incorporate sound and movement to emphasize the refreshing nature of water and enhance simulations in response to advertisements. In addition, future research could more closely examine the influence of packaging color or shape of bottled water in guiding representations of reward and subsequent consumption motivation [54, 55].

While the consistent indirect effects reported are promising, the absence of main effects of advertising condition on our measures of motivation suggests that the strategy to increase the appeal of water through simulations of consumption and enjoyment can still be further improved. For example, it is possible that designing interventions that directly engage people with their own rewarding consumption representations, such as through directed questions, may be even more effective, or that interventions that allow people to consolidate representations in terms of enjoyment through drinking cool, refreshing water while exposed to an intervention may produce stronger results. Overall, we would encourage researchers and practitioners in this area to consider the powerful mechanism of simulations that can drive motivation, and to develop creative ways of tapping into this mechanism that may work for the particular target group of a planned intervention.

Importantly, while advertisements emphasizing the health benefits of water shifted representations of water toward being seen as beneficial in the long-term, which in turn affected the extent to which participants reported simulating a rewarding consumption experience, this effect was significantly smaller than the effect of simulation-enhancing advertisements on motivation through increased representations of consumption and reward. Future studies could test whether emphasizing both properties of bottled water is even more effective in increasing motivation than focusing on either dimension alone.

The finding that the increase in motivation through health perceptions may be driven by an increase in simulations of drinking the water may warrant further research. These findings suggest that health-focused advertisements may also boost representations of consumption and reward, or activate simulations of drinking it, and thus increase motivation. In this case, perceiving water as healthier may influence perceptions of how tasty the water is, or how rewarding it will be to consume the water. This is consistent with previous research suggesting that health and taste perceptions are closely related [39, 40] and that emphasizing the rewarding aspects of healthy foods in language increases the desire to consume them in that moment [18, 19]. Whether this effect holds for foods and drinks that are more or less appealing than the rather neutral product water, may be an interesting question for future studies. Again, this finding points to the important and potentially underutilized role of consumption and reward simulations for promoting health behaviors.

Finally, although we only tested this approach with respect to bottled water in an advertising context, we suggest that the underlying principle of emphasizing consumption and reward experiences, and thus the immediate benefits of consumption, may also apply to tap water, which is more affordable and sustainable as a beverage than bottled water.

### Strengths and limitations

This series of experiments is the first to examine the impact of advertising on cognitive representations of bottled water in relation to motivation. Given the negative public health effects of SSBs and the strong industry and lobby supporting them [56, 57], it is crucial to increase the attractiveness of healthier beverages. Our results show that using consumption and reward language and visual imagery as typically employed in SSB campaigns [57], can change people’srepresentation of bottled water, which in turn can increase motivation to consume it, more than by emphasizing the health benefits of consuming water. These findings were replicated across three experiments with large samples and pre-registered hypotheses. Consistent findings were observed across several measures, in particular desire and WTP, with our Feature Listing task potentially being less susceptible to demand effects than traditional rating scales, supporting the robustness of these findings.

Nevertheless, several limitations warrant consideration. First, all experiments were conducted online and did not involve an actual choice or purchase task. Future studies should replicate this paradigm in a real-life consumer setting. Second, our sample consisted of regular water drinkers. Across experiments, participants reported consuming tap water 2.3 times more often and bottled water 1.7 times more often than SSBs. This is higher than population-level measures of water and SSB consumption [58]. While this is encouraging, as it suggests that even in a sample of regular water drinkers, our advertisements induced a representational shift and increased motivation for a bottled water, future studies could explore the effectiveness of such advertisements among infrequent water consumers.

Finally, although the advertisements induced representational shifts captured during the experiments, the duration of these shifts and their potential long-term effects on motivation remain to be tested. Previous research suggests that advertisements can shape expectations and actual sensory experiences of food [20, 59]. Future studies could examine how expectations regarding the taste or reward value of water interact with the actual experience while consuming water.

## Conclusion

This research suggests that viewing advertisements for bottled water can shift representations of the water and increase motivation to consume it. Three experiments showed that advertisements emphasizing the rewarding or health aspects of a fictitious bottled water shifted cognitive representations of a bottled water, at least temporarily. The extent to which these representations included consumption and reward features, or positive health consequences, in turn, predicted increased desire and WTP for the bottled water. Specifically, viewing advertisements that emphasized the sensory and consumption aspects of water increased the degree to which participants represented the water in terms of drinking and enjoying it, which in turn increased motivation to drink the water. These findings suggest that emphasizing the immediate consumption rewards of water drinking in health interventions or campaigns may be more effective at instigating health behavior change than merely emphasizing the health benefits of water.

### Electronic supplementary material

Below is the link to the electronic supplementary material.

Supplementary Material 1

## Data Availability

The datasets supporting the conclusions of this article are available in the OSF repository: https://osf.io/s4kwv/?view_only=9ef5c3ebba424b73b153a018744fcc0b.
