# Supplementary material for: Representational shifts: increasing motivation for bottled water through simulation-enhancing advertisements
Source: BMC Public Health. 2023 Nov 9;23:2209. doi: 10.1186/s12889-023-17109-1 (PMC10634071; doi:10.1186/s12889-023-17109-1)
Supplement: Supplementary file 1 — Supplementary Material 1 [file 12889_2023_17109_MOESM1_ESM.pdf]

Supplemental Online Material

In this document we present methodological details and supplemental analyses for the paper *Representational shifts: Increasing motivation for bottled water through advertisements.* Analyses files and other complementary materials can be found on our repository on the OSF: https://osf.io/s4kwv/?view_only=9ef5c3ebba424b73b153a018744fcc0b.

ANOVA analyses 2
Adjusted alpha levels to control for Type 1 error 2
Experiment 1 ***3***
Advertisements used in Experiment 1 3
Language used in the Feature Listing Task across advertisement conditions 4
Percentage of features generated for each advertisement condition 6
Mediation through shifts in cognitive representations 6
Testing covariates 8
Moderated mediation of the indirect effect through consumption and reward 10
Experiment 2 ***11***
Pilot for selecting health-focused advertisements for Exp. 2 11
Advertisements used in Experiment 2 13
Percentage of features generated for each advertisement condition 14
Serial mediation 14
Testing covariates 15
Robustness check for serial mediation 17
Experiment 3 ***18***
Advertisements used in Experiment 1 18
Percentage of features generated for each advertisement condition 19
Testing covariates 20
Robustness check for serial mediation 21

ANOVA analyses

Here we report the results of ANOVA analyses examining the overall effect of condition on our dependent variables and exploratory variables anticipated reward, anticipated health benefits, attractiveness, desire and WTP (see Table S1). In the manuscript we report the results of *t*-tests comparing differences between conditions given that these were pre-registered in our analysis plan. None of the t-tests were significant were the F-test was not so the results from both types of analyses are consistent with each other.

Table S1

Test statistics of ANOVA tests examining the effect of condition on the variables measured in Exp. 1-3.

**Experiment 1**	Mean square	*df*	*F*	*p*
Anticipated reward	745	2	1.75	.174
Anticipated health	3320	2	10.2	< .001
Attractiveness	264	2	0.35	.704
Desire	621	2	0.70	.495
WTP	0.06	2	0.70	.496
**Experiment 2**				
Anticipated reward	358	2	0.83	.438
Anticipated health	1860	2	5.79	.003
Desire	133	2	0.16	0.855
WTP	0.17	2	1.68	0.188
**Experiment 3**				
Anticipated reward	343	2	0.85	.429
Anticipated health	409	2	1.30	.272
Desire	81.3	2	0.10	.905
WTP	0.14	2	1.26	.283

Adjusted alpha levels to control for Type 1 error

We adjusted alpha levels to control for condition contrasts and multiple testing in our confirmatory analyses. More specifically, we made the following adjustments.

Exp.1: Main effects: 0.05/2 → 0.025/6 tests = 0.004; Mediation: 0.05

Exp.2: Main effects: 0. 05/2 condition contrasts → 0.025/2 tests = 0.0125; Mediation: 0.05/2 = 0.025

Exp.3: Main effects: 0. 05/2 condition contrasts → 0.025/2 tests = 0. 0125; Mediation: 0.05/2 = 0.025

Experiment 1

Advertisements used in Experiment 1

Fig. S1

The three slogans and image descriptions for each advertisement condition in Exp. 1. The included images are examples of what these advertisements looked like. The actual advertisements used can be requested from the authors.

<table>
<tr><td rowspan="2">Simulation-enhanced</td><td>Slogan</td><td>INVIGORATE YOUR BODY WITH A COOL SPLASH OF AQUAVIVA</td><td>FEEL AND SHARE THE FRESH ENERGY OF SUMMER</td><td>REFRESH ALL YOUR SENSES WITH THIS SMOOTH, COOL WATER</td><td>Examples</td></tr>
<tr><td>Image description</td><td>A family playing in a blow-up pool in the garden with water splashing around.</td><td>A group of friends running into the sea and splashing water.</td><td>A man and woman enjoying drinking water from Aqauviva water bottles after exercising.</td><td rowspan="3">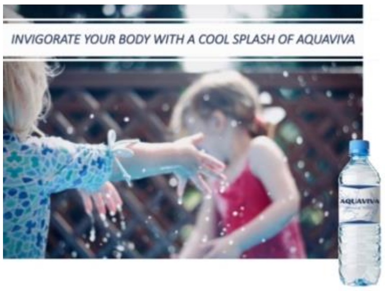
</td></tr>
<tr><td rowspan="2">Health-focused</td><td>Slogan</td><td>AQUAVIVA TAKES CARE OF YOUR HEALTH</td><td>THE PUREST GIFT FOR HEALTHY BODIES</td><td>STAY YOUNG WITH THIS PURIFYING WATER</td></tr>
<tr><td>Image description</td><td>A family playing in a blow-up pool in the garden with water splashing around.</td><td>A group of friends running into the sea and splashing water.</td><td>A man and woman enjoying drinking water from Aqauviva water bottles after exercising.</td></tr>
<tr><td rowspan="2">Control</td><td>Slogan</td><td>WITH US YOU SAVE EASILY AND HAPPILY</td><td>KEEP YOUR BELONGINGS IN SAFE HANDS</td><td>100% HASSLE-FREE: WE DO THE WORK, YOU ENJOY</td><td rowspan="2">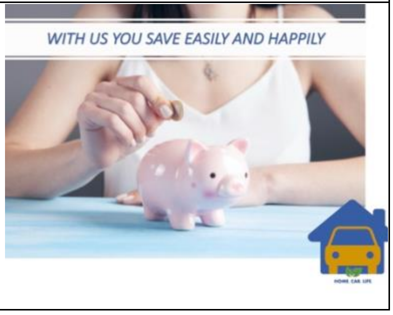
</td></tr>
<tr><td>Image description</td><td>A woman putting a coin in a piggy bank.</td><td>A couple unpacking boxes during a house move.</td><td>A woman on holiday lounging in a convertible.</td></tr>
</table>

Language used in the Feature Listing Task across advertisement conditions

An alternative explanation for our findings is that participants describe the bottled water in the Feature Listing (FL) task using the words they have read in the advertisements. In order to rule out this explanation, we compared the features listed by participants across the conditions.

Looking at the words used in the advertisements, simulation-enhancing advertisements included the words “invigorate”, “cool splash”, “fresh energy”, “refresh senses”, “smooth”, “cool” while health-focused advertisements included “takes care”, “health(/y)”, “purest”, “stay young”, “purifying”. Control advertisements did not contain any words related to the bottled water.

Participants used a large variety of words to describe the bottled water, ranging between 376 and 419 unique features. Moreover, looking at the overlap between features used in the conditions, we find that 28% of features used were used across all three conditions and approximately another 10% of features overlapped between sets of conditions, leaving 10-18% of features unique to specific conditions (See Fig. S2).

Fig. S2

Venn diagram showing the unique and overlapping features listed by participants in the Feature Listing task across the three difference conditions.

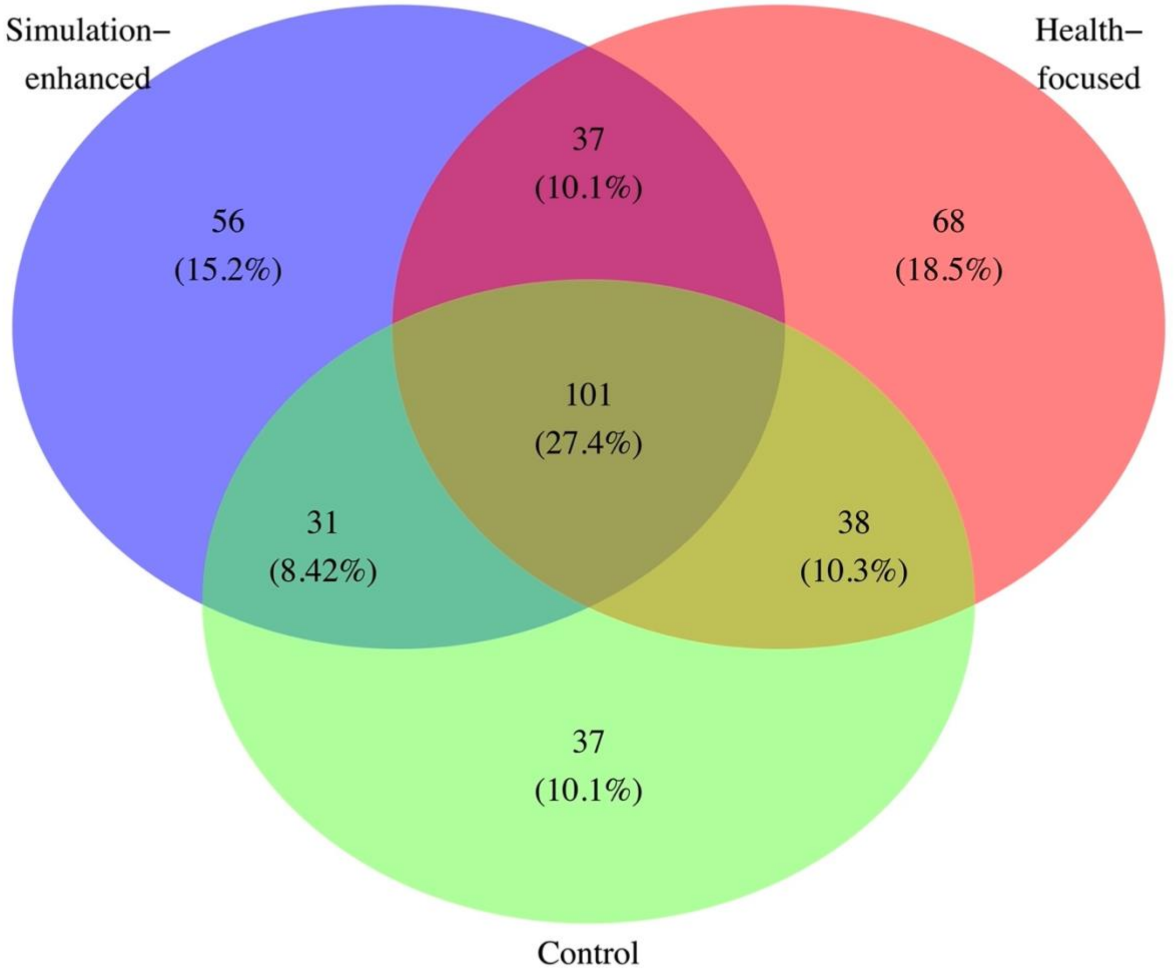

In addition, the word that was most often used across all conditions did not feature in the advertisement. The word “refreshing” was the most often used and made up 12% of all the words used in the simulation-enhancing advertisement condition, and 9% in the health-focused and control advertisement condition. These observations suggests that participants relied on language associated to their representations of the bottled water rather than merely copying the words used in the advertisements.

Percentage of features generated for each advertisement condition

Fig. S3

Percentage of features for bottled water for the categories non-consumption situation, situation-independent, and consumption situation

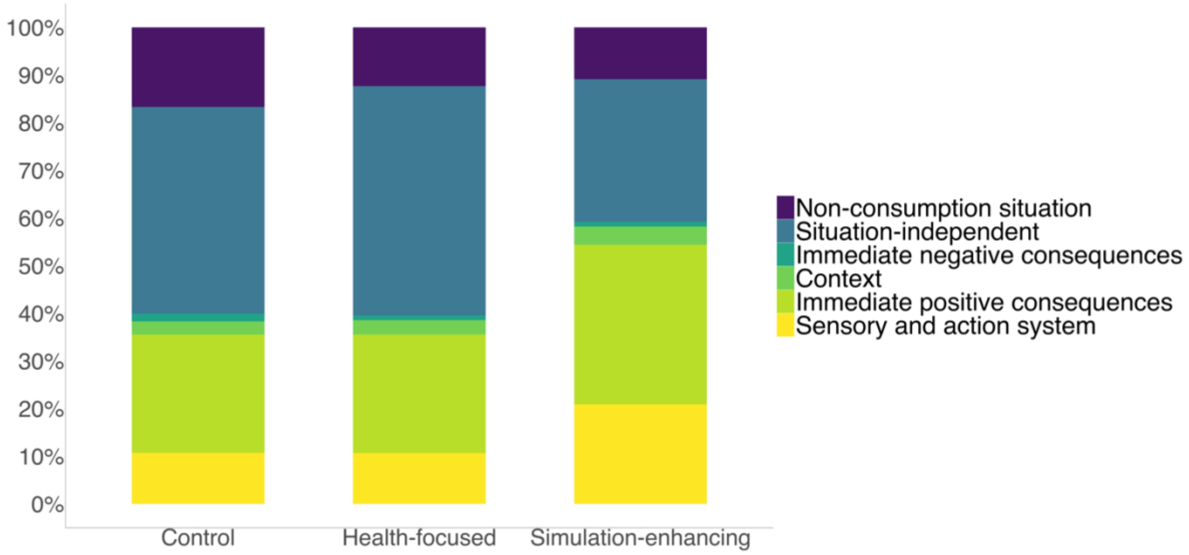

Note. Consumption and reward features include features related to context, immediate positive consequences, and sensory and action system.

Mediation through shifts in cognitive representations

See p3_exp2.html for precise results of mediation analyses.

Fig. S4

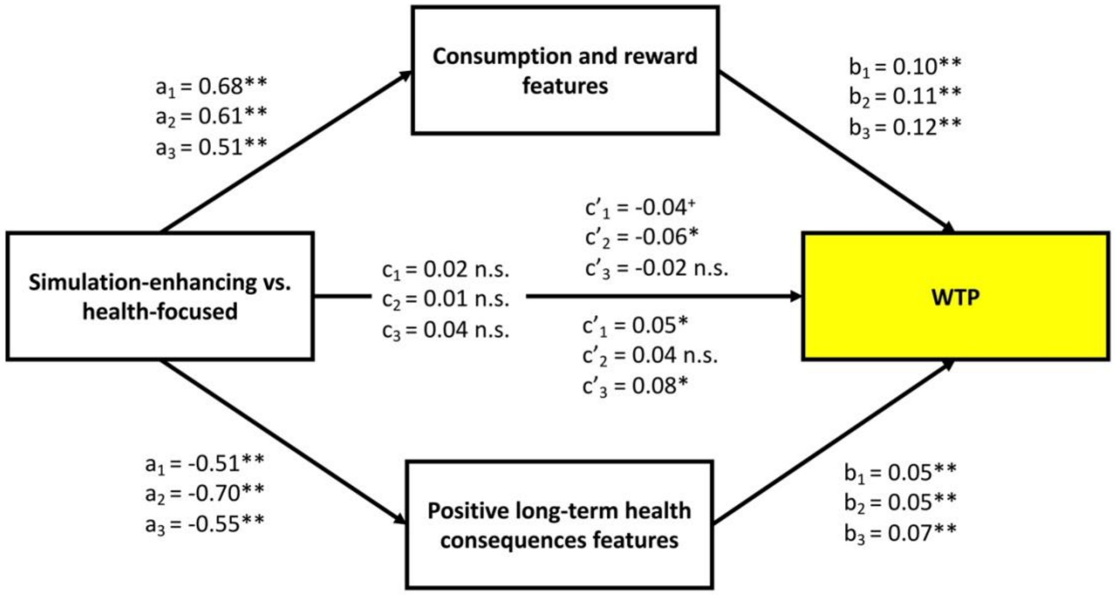

Note. $^{**}p < 0.001$, $^{*}p < 0.05$, $^{+}p < 0.10$, n.s. = not significant

Testing covariates

We assessed stepwise whether the covariates had an impact on the influence of advertisement condition on the dependent variables. We first examined the main effect (step 1) and then entered the interaction effect (step 2) into the model. If an interaction was significant, we explored it in further analyses.

				**Covariates**					
**DV**	**Label Type**		Thirst	Bottled water frequency	Tap water frequency	SSB frequency	BMI	Gender	Age
Consumption and reward features	Enhancing vs. health	Main effect							
		Interaction							
	Enhancing vs. control	Main effect							
		Interaction							
	Health vs. control	Main effect							
		Interaction							
Anticipated reward	Enhancing vs. health	Main effect							
		Interaction							
	Enhancing vs. control	Main effect							
		Interaction		*p* = .051		*p* = .010			
	Health vs. control	Main effect							
		Interaction					*p* = .007		
Attractive-ness	Enhancing vs. health	Main effect							
		Interaction							
	Enhancing vs. control	Main effect							
		Interaction							
	Health vs. control	Main effect							
		Interaction							
Desire	Enhancing vs. health	Main effect							
		Interaction							
	Enhancing vs. control	Main effect							
		Interaction							
	Health vs. control	Main effect							
		Interaction							
Choice	Enhancing vs. health	Main effect							
		Interaction							
	Enhancing vs. control	Main effect							
		Interaction							
	Health vs. control	Main effect							
		Interaction							

Three interactions effects were significant:

- Frequency of bottled water consumption moderated the effect of control compared to simulation-enhancing advertisements on anticipated reward, $b = -2.99$, $SE = 1.53$, $p = .051$. Examining the simple effects of advertisement for infrequent (-1 *SD* below the mean), average (mean), and frequent consumers of bottled water consumers (+1 *SD* above the mean), showed that the effect of advertisement on anticipated reward was only significant for infrequent ($b = 6.09$, $SE = 2.17$, $p = .010$) and average consumers of bottled water ($b = 3.09$, $SE = 1.53$, $p = .040$), but not for frequent consumers of bottled water ($p = .960$).
- Frequency of SSB consumption moderated the effect of control vs. simulation-enhancing advertisements on anticipated reward, $b = 4.17$, $SE = 1.62$, $p = .010$. Examining the simple effects of advertisement for infrequent (-1 *SD* below the mean), average (mean), and frequent SSB consumers (+1 *SD* above the mean), showed that the effect of advertisement on anticipated reward was only significant for frequent ($b = 7.13$, $SE = 2.30$, $p < .001$), almost for average ($b = 2.95$, $SE = 1.63$, $p = .070$), but not for infrequent SSB consumers ($p = .590$).
- BMI moderated the effect of control compared to simulation-enhancing advertisements on anticipated reward, $b = 4.31$, $SE = 1.59$, $p = .007$. Examining the simple effects of advertisement for participants with lower (-1 *SD* below the mean), average (mean), and higher BMI (+1 *SD* above the mean), showed that the effect of advertisement on anticipated reward was only significant for those with higher BMI ($b = 5.54$, $SE = 2.29$, $p = .020$) but not for those with average ($p = .480$) or low BMI ($p = .160$).

Moderated mediation of the indirect effect through consumption and reward

We also explored whether thirst, frequency of bottled water and SSB consumption moderated the indirect effects of advertisement through consumption and reward features and positive long-term health consequences on attractiveness (moderated mediation), and this was not the case.

Experiment 2

Pilot for selecting health-focused advertisements for Exp. 2

We selected eight images involving people in situations similar to those used in the advertisements in Exp. 1 such as a family having a picknick in the parc or a group of people doing yoga. We choose images that could be paired with the idea of water as healthy but not refreshing. Each of these images was paired with the three slogans that will be used in this experiment. Thus, there were a total of 24 new health-focused advertisements, three simulation-enhancing advertisements and three control advertisements to be rated, totalling 30 advertisements.

We recruited a sample of $N = 200$ participants from Prolific who rated 50% of the advertisements as well as 50% of the 14 individual images used in the advertisements. Thus, they were each shown 7 images and 15 advertisements and were asked to evaluate the valence of the image: “I find this image…” with the anchors -50 = *negative* to 50 = *positive*. They were also asked to evaluate the advertisements on the following questions: “To what extent can you imagine being in the situation shown in the advertisement?” with the anchors 0 = *not at all,* 100 = *totally*, and “I find that the slogan fits the image…” with the anchors 50 = *negative* to 50 = *positive.* Whether they first rated the images or the advertisements was counterbalanced across participants.

Comparing the three ratings for simulation-enhancing advertisements and the control advertisements used in Exp. 1, we found that simulation-enhancing ads were rated more positively, were easier to engage in, and had slogans that were rated as more fitting with the images, compared to control advertisements. Thus, we will control for these advertisement characteristics in Exp. 2.

Because we were most interested in the comparison between simulation-enhancing and health-focused advertisements, we chose to select the health-focused advertisements that

best matched the simulation-enhancing ads and not the control ads. To do this we employed a and selected the health-focused ads that were least distant in terms of the three ratings. See Table S2 for an overview of the ratings per advertisements.

Table S2

Mean advertisement ratings of valence, fitting, and imagining.

		**Valence**	**Fits**	**Imagining**
		M (*SD*)		
**Control advertisements**	1	72.1 (24.1)	62.8 (27.4)	47.7 (28.7)
	2	68.5 (19.4)	57.7 (25.8)	63.1 (23.6)
	3	70.0 (23.4)	61.2 (26.7)	46.4 (26.7)
		70.2 (22.4)	60.6 (26.6)	52.4 (27.4)
**Simulation-enhancing advertisements**	1	86.1 (15.7)	63.4 (29.0)	51.9 (30.1)
	2	88.8 (16.6)	73.0 (21.4)	66.4 (25.6)
	3	74.2 (18.7)	70.2 (26.7)	58.6 (29.8)
		82.9 (18.1)	68.8 (26.1)	59.0 (29.1)
**Health-focused advertisements**	1	80.5 (15.5)	57.7 (26.3)	56.3 (29.5)
	2	83.8 (15.9)	66.3 (23.2)	67.8 (23.5)
	3	76.1 (22.2)	67.2 (21.2)	69.3 (25.1)
		80.1 (18.4)	63.7 (24.0)	64.5 (26.7)

Note. All ratings were scaled to range from 0 to 100.

Advertisements used in Experiment 2

Fig. S5

The three slogans and image descriptions for each advertisement condition in Exp. 2. The included images are examples of what these advertisements looked like. The actual advertisements used can be requested from the authors.

Simulation-enhanced	Slogan	INVIGORATE YOUR BODY WITH A COOL SPLASH OF AQUAVIVA	FEEL AND SHARE THE FRESH ENERGY OF SUMMER	REFRESH ALL YOUR SENSES WITH THIS SMOOTH, COOL WATER	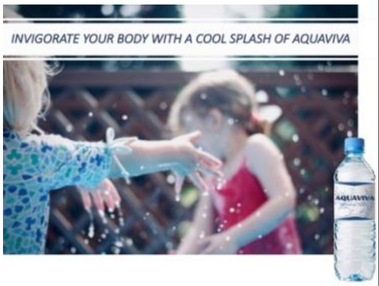
	Image description	A family playing in a blow-up pool in the garden with water splashing around.	A group of friends running into the sea and splashing water.	A man and woman enjoying drinking water from Aqauviva water bottles after exercising.	
Health-focused	Slogan	AQUAVIVA TAKES CARE OF YOUR HEALTH	STAY YOUNG WITH THIS NOURISHING WATER	THE HEALTHIEST GIFT FOR ALL-ROUND VITALITY	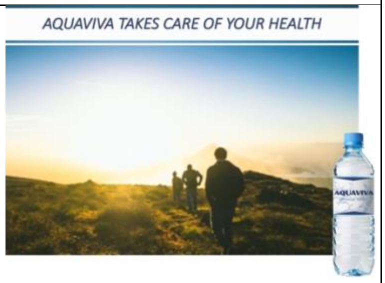
	Image description	A couple toasting with Aquaviva water bottles during a hike.	A man and woman stretching in the grass after running.	Three friends hiking.	
Control	Slogan	WITH US YOU SAVE EASILY AND HAPPILY	KEEP YOUR BELONGINGS IN SAFE HANDS	100% HASSLE-FREE: WE DO THE WORK, YOU ENJOY	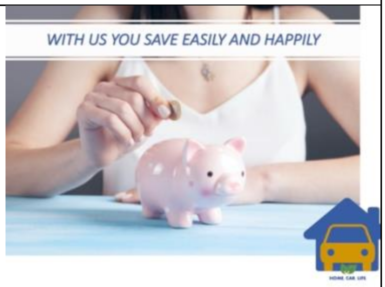
	Image description	A woman putting a coin in a piggy bank.	A couple unpacking boxes during a house move.	A woman on holiday lounging in a convertible.	

Percentage of features generated for each advertisement condition

Fig. S6

Percentage of features for bottled water for the categories non-consumption situation, situation-independent, and consumption situation

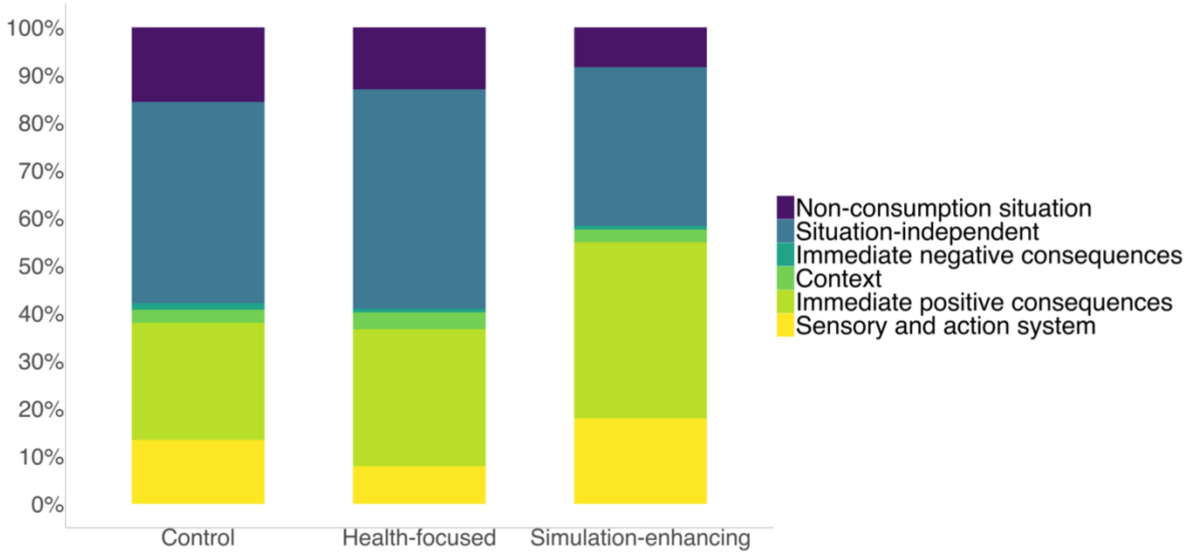

Note. Consumption and reward features include features related to context, immediate positive consequences, and sensory and action system.

Serial mediation

See p3_exp2.html for precise results of mediation analyses.

Fig. S7

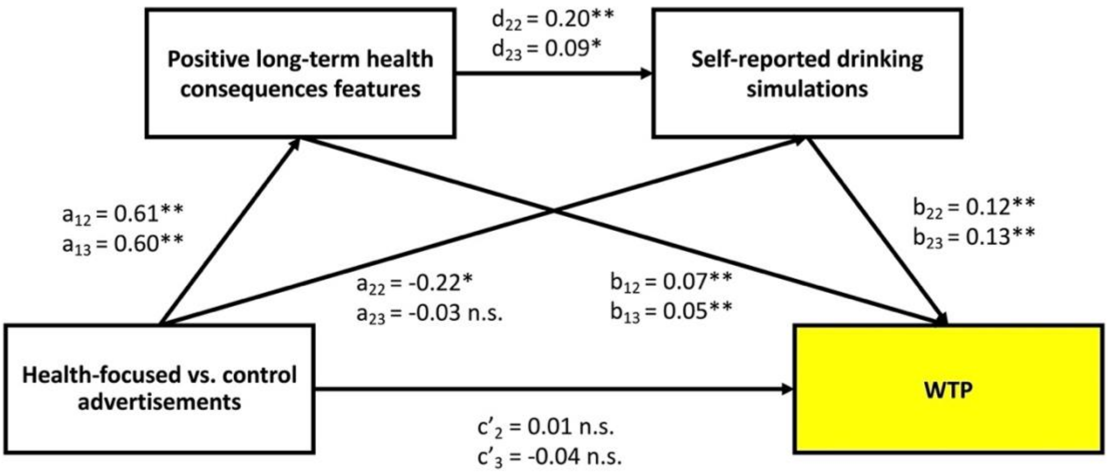

Testing covariates

We assessed stepwise whether the covariates had an impact on the influence of advertisement condition on the dependent variables. We first examined the main effect (step 1) and then entered the interaction effect (step 2) into the model. If an interaction was significant, we explored it in further analyses.

				Covariates					
**DV**	**Label Type**		Thirst	Bottled water frequency	Tap water frequency	SSB frequency	BMI	Gender	Age
Consumption and reward features	Enhancing vs. health	Main effect							
		Interaction							
	Enhancing vs. control	Main effect							
		Interaction							
	Health vs. control	Main effect							
		Interaction							
Positive long-term health consequences features	Enhancing vs. health	Main effect							
		Interaction							
	Enhancing vs. control	Main effect							
		Interaction							
	Health vs. control	Main effect							
		Interaction							
Desire	Enhancing vs. health	Main effect							
		Interaction							
	Enhancing vs. control	Main effect							
		Interaction							
	Health vs. control	Main effect							
		Interaction							
WTP	Enhancing vs. health	Main effect							
		Interaction							
	Enhancing vs. control	Main effect							
		Interaction						*p* = .005	
	Health vs. control	Main effect							
		Interaction						*p* < .001	

Two interactions effects were significant:

- Gender moderated the effect of control compared to simulation-enhancing advertisements on WTP, $b = 0.17$, $SE = 0.06$, $p = .005$; $b = 0.20$, $SE = 0.06$, $p < .001$. Examining the simple effects of advertisement for female vs. male participants, showed that simulation-enhancing advertisements did not affect WTP compared to

control advertisements for men, *b* = -0.07, *SE* = 0.05, *p* = .17, but did increase WTP for women, *b* = 0.10, *SE* = 0.03, *p* < .01.

- In addition, gender moderated the effect of control compared to health-focused advertisements on WTP, *b* = 0.20, *SE* = 0.06, *p* < .001. Examining the simple effects of advertisement for female vs. male participants, showed that health-focused advertisements increased WTP compared to control advertisements for females, *b* = 0.11, *SE* = 0.03, *p* < .001, but decreased WTP for men, *b* = -0.10, *SE* = 0.05, *p* = .04.

Robustness check for serial mediation

Table S3

Results of robustness check for the serial mediation in Experiment 2.

	Pathway tested	Estimate indirect effect	Comments
**Reverse mediation 1**	Health-focused vs. control advertisements → drinking simulations → proportion of long-term positive health consequences → **desire**	b = -0.06, SE = 0.06, p = .303, 95% CI [-0.18, 0.06]	Reverse mediation is not significant.
	Health-focused vs. control advertisements → drinking simulations → proportion of long-term positive health consequences → **WTP**	b = -0.01, SE = 0.01, p = .282, 95% CI [-0.01, 0.01]	
**Alternative mediator 1**	Health-focused vs. control advertisements → proportion of long-term positive health consequences → anticipated reward → **desire**	b = 3.14, SE = 0.71, p < .001, 95% CI [1.74, 4.54]	The same indirect effect is significant when anticipated reward is taken as a mediator. This suggests that this measure captures similar variance as that by our measure of self-reported drinking simulations. As predicted by the grounded cognition theory of motivation, drinking simulations predict reward.
	Health-focused vs. control advertisements → proportion of long-term positive health consequences → anticipated reward → **WTP**	b = 0.02, SE = 0.01, p < .001, 95% CI [0.01, 0.03]	
**Other condition contrast**	Simulation-enhancing vs. control advertisements → drinking simulations → proportion of long-term positive health consequences → **desire**	b = -0.14, SE = 0.15, p = .339, 95% CI [-0.43, 0.15]	The effect is dependent on the health-focused condition.
	Simulation-enhancing vs. control advertisements → drinking simulations → proportion of long-term positive health consequences → **WTP**	b = -0.01, SE = 0.01, p = .340, 95% CI [-0.01, 0.01]	

Experiment 3

Advertisements used in Experiment 1

Fig. S8

The three slogans and image descriptions for each advertisement condition and filler items in Exp. 3. The included images are examples of what these advertisements looked like. The actual advertisements used can be requested from the authors.

Simulation-enhanced	Slogan	INVIGORATE YOUR BODY WITH A COOL SPLASH OF AQUAVIVA	FEEL AND SHARE THE FRESH ENERGY OF SUMMER	REFRESH ALL YOUR SENSES WITH THIS SMOOTH, COOL WATER	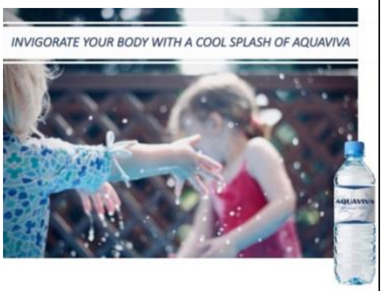
	Image description	A family playing in a blow-up pool in the garden with water splashing around.	A group of friends running into the sea and splashing water.	A man and woman enjoying drinking water from Aqauviva water bottles after exercising.	
Health-focused	Slogan	AQUAVIVA TAKES CARE OF YOUR HEALTH	STAY YOUNG WITH THIS NOURISHING WATER	THE HEALTHIEST GIFT FOR ALL-ROUND VITALITY	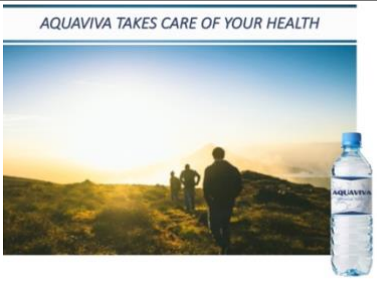
	Image description	A couple toasting with Aquaviva water bottles during a hike.	A man and woman stretching in the grass after running.	Three friends hiking.	
Control	Slogan	-	-	-	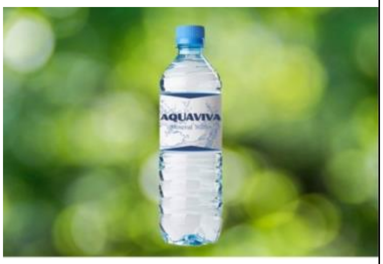
	Image description	An Aquaviva bottle on a picnic table with green trees in the background.	An Aquaviva bottle on a picnic table with green trees in the background.	An Aquaviva bottle on a picnic table with green trees in the background.	

Percentage of features generated for each advertisement condition

Fig. S9

Percentage of features for bottled water for the categories non-consumption situation, situation-independent, and consumption situation

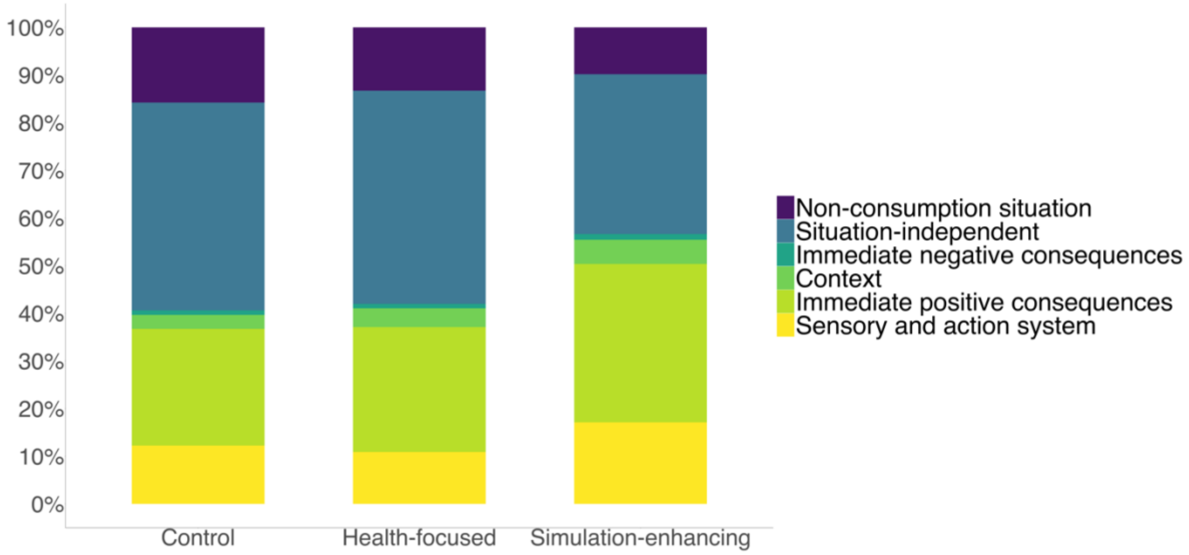

Note. Consumption and reward features include features related to context, immediate positive consequences, and sensory and action system.

Testing covariates

We assessed stepwise whether the covariates had an impact on the influence of advertisement condition on the dependent variables. We first examined the main effect (step 1) and then entered the interaction effect (step 2) into the model. If an interaction was significant, we explored it in further analyses.

				**Covariates**						
**DV**	**Label Type**		Thirst	Bottled water frequency	Tap water frequency	SSB frequency	BMI	Gender	Age
Consumption and reward features	Enhancing vs. health	Main effect							
		Interaction							
	Enhancing vs. control	Main effect							
		Interaction							
	Health vs. control	Main effect							
		Interaction							
Positive long-term health consequences	Enhancing vs. health	Main effect							
		Interaction							
	Enhancing vs. control	Main effect							
		Interaction							
	Health vs. control	Main effect							
		Interaction							
Desire	Enhancing vs. health	Main effect							
		Interaction							
	Enhancing vs. control	Main effect							
		Interaction							
	Health vs. control	Main effect							
		Interaction							
WTP	Enhancing vs. health	Main effect							
		Interaction					p = .014		
	Enhancing vs. control	Main effect							
		Interaction							
	Health vs. control	Main effect							
		Interaction							

One interaction was significant:

BMI moderated the effect of health-focused compared to simulation-enhancing advertisements on WTP, b = -0.06, SE = 0.03, p = .014. Examining the simple effects of advertisement for people with low (-1 *SD* below the mean), average (mean), and high (+1 *SD*

above the mean) BMI, showed that the effect of advertisement on WTP was only significant for those with low BMI, $b = 0.11$, $SE = 0.04$, $p = .010$.

Robustness check for serial mediation

Table S4

Results of robustness check for the serial mediation in Experiment 3.

	Pathway tested	Estimate indirect effect	Comments
**Reverse mediation 1**	Health-focused vs. control advertisements → drinking simulations → proportion of long-term positive health consequences → **desire**	$b = 0.01$, $SE = 0.01$, $p = .799$, 95% CI [-0.02, 0.02]	Reverse mediation is not significant.
	Health-focused vs. control advertisements → drinking simulations → proportion of long-term positive health consequences → **WTP**	$b = 0.00$, $SE = 0.00$, $p = .797$, 95% CI [-0.01, 0.01]	
**Alternative mediator 1**	Health-focused vs. control advertisements → proportion of long-term positive health consequences → anticipated reward → **desire**	$b = 1.86$, $SE = 0.54$, $p < .001$, 95% CI [0.80, 2.93]	The same indirect effect is significant when anticipated reward is taken as a mediator. This suggests that this measure capture similar variance as that by our measure of self-reported drinking simulations. As predicted by the grounded cognition theory of motivation, drinking simulations predict reward.
	Health-focused vs. control advertisements → proportion of long-term positive health consequences → anticipated reward → **WTP**	$b = 0.02$, $SE = 0.01$, $p < .001$, 95% CI [0.01, 0.02].	
**Alternative condition contrast**	Simulation-enhancing vs. control advertisements → drinking simulations → proportion of long-term positive health consequences → **desire**	$b = 0.20$, $SE = 0.26$, $p = .438$, 95% CI [-0.30, 0.70].	The effect is dependent on the health-focused condition.
	Simulation-enhancing vs. control advertisements → drinking simulations → proportion of long-term positive health consequences → **WTP**	$b = 0.01$, $SE = 0.01$, $p = .439$, 95% CI [-0.01, 0.01].	
